# The mediating effect of allostatic load on the association between life course socioeconomic disadvantage and chronic pain: a prospective finding from the National Survey of Midlife Development in the United States

**DOI:** 10.3389/fpain.2023.1213750

**Published:** 2023-07-13

**Authors:** Yunlong Liang

**Affiliations:** Institute for Social and Economic Research, University of Essex, Colchester, United Kingdom

**Keywords:** life course socioeconomic disadvantages, chronic pain, allostatic load, biomarkers, mediation analysis, resilience

## Abstract

**Background:**

Socioeconomic disadvantages (SEDs) are associated with chronic pain (CP) and allostatic load (AL). Few prospective population-based studies have examined the relationship between life course SED, CP interference, and CP widespreadness, and there is no prospective population-based study on whether AL mediates the association between SED and CP.

**Objective:**

In this study, we investigated whether the prospective effect of SED on CP at Midlife in the United States (MIDUS) 3 is consistent with the accumulation of risk model and social mobility model, using the National Survey of MIDUS (*n* = 593). To prepare for the mediation analysis, we tested (1) whether SED would be prospectively associated with AL in the MIDUS 2 biomarker project, (2) whether AL would be prospectively associated with CP, and (3) whether childhood, as a critical period, moderated the association between AL and CP. In addition, the mediating effect of AL on the association between SED and CP was examined.

**Method:**

SED was measured using cumulative scores and disadvantage trajectories derived from latent class trajectory modeling (LCTM). After multiple imputations, analyses were conducted using multinomial logistic regression for CP and negative binomial regression for AL, respectively. Finally, mediation analyses and moderated mediation analyses were performed.

**Results:**

LCTM identified three SED trajectories, namely, constant low, high to low, and medium to high. The results showed that proximal cumulative SED was associated with high-interference CP. Furthermore, compared with the group with constant low SED, the group with medium-to-high SED was significantly associated with high-interference pain and experienced pain in at least three different sites. Cumulative SED and deteriorating SED trajectories were associated with higher AL, consistent with previous studies. Furthermore, childhood SED moderated the effect of AL on CP widespreadness and unexpectedly demonstrated a protective effect, while other associations between AL and CP were not significant. Subsequent mediation analysis did not yield statistically significant evidence.

**Conclusions:**

People who experienced more recent SED or increasing disadvantage throughout their lives were more likely to suffer from CP, and this association was not mediated by physiological system dysregulation caused by chronic stress. Therefore, measures to alleviate AL may not be effective in protecting socioeconomically disadvantaged populations from CP.

## 1. Introduction

The study of the relationship between socioeconomic dislocation and individual anomic behavior and experiences can be traced back to the inception of sociology (1). Over the past half-century, the American socioeconomic structure has undergone a gradual decline concomitant with the process of deindustrialization and continuous trade disadvantages, which has precipitated a surge in anomic phenomena known as “deaths of despair” (2), which encompass suicides, drug overdoses, and alcoholism deaths among middle-age individuals (3). Coincidentally, the prevalence of chronic pain (CP) among Americans has also increased during the same period (4–7). The conjugate trend underscores the necessity of examining the relationship between socioeconomic disadvantages (SEDs) and CP because CP may not only serve as the intermediary mechanism connecting socioeconomic collapse with the deaths of despair (8, 9) but also play a pivotal role in engendering a more extensive public burden (10). The International Association for the Study of Pain (IASP) defines CP as pain that persists or recurs for more than 3 months (11). A meta-analysis indicates that CP affects approximately 43.5% of the general population in the United Kingdom (12). In the United States, approximately 20.5% of adults suffer from CP each year, which causes substantial burdens on the national health system as well as a considerable productivity loss of over $296 billion (10).

CP is suggested to be a crucial factor in suicides related to deaths of despair (8). Both CP and deaths of despair seem to share an antecedent cause—protracted socioeconomic turmoil. SED reflects an individual's or a group's lower position in the social hierarchy, characterized by their limited economic and social resources, and it encompasses facing economic hardships, social inequality, and reduced access to resources and opportunities compared with more advantaged social groups (13, 14). For decades, SED has been identified as the fundamental cause of chronic disease disparities between different social groups (13). The fundamental cause theory posits that the unequal distribution of flexible resources among individuals is the main driving force for the varying vulnerability of distinct social groups to diseases (13). The accumulation of risk model further links life course SED to health disparities (15). Individuals who consistently face SED are at a heightened risk of exposure to detrimental stressors, or SED can be considered the persistent stressor itself (16), increasing the probability of experiencing CP. Furthermore, the social mobility framework integrates the temporal variations in the level of SED experienced during different stages of life into the accumulation of risk model. The social mobility model elucidates the progression of individuals as they move up or down the social class ladder throughout their entire life span (17, 18). Recent studies have corroborated the trajectory of SED on health outcomes: descending SED is positive for one's wellbeing (19, 20), and the detrimental effect of early-life adversity can be alleviated or even reversed by upward social mobility (21, 22).

A previous topical review has revealed substantial evidence of a statistically significant association between SED and an increased prevalence of CP, elevated levels of pain interference, and a greater extent of CP widespreadness (23). Few cohort studies prospectively examined the differential impacts of life course SEDs on CP widespreadness (24–26) and the recent cohort studies have several limitations. First, only a few population-based prospective studies have investigated the association between SED and pain interference. Similar to the number of pain sites, pain interference is closely linked to functional limitations in patients in pain (27), and such limitations provoke physical, psychological, and societal issues in an individual’s life (28). Thus, it is necessary to incorporate pain interference and the number of pain sites in the examination. Second, chronic illnesses have not been considered a substantial confounder influencing SED (29) and CP (30). In addition, although population-based research on social mobility and low back pain (pain lasting 7 days or more in the past 12 months) exists, the study did not utilize the definition of CP in terms of its duration (31). Therefore, the trajectories of SED link to CP remain unknown. Finally, previous studies have explored the mediation mechanisms of psychological distress (25) and obesity (26), but how SEDs are prospectively linked with CP through sociobiological mechanisms remains unknown.

Allostatic load (AL) is defined as the physiological wear and tear arising from repeated adaptations to chronic stressors (32). It begins with the prolonged activation of the hypothalamus–pituitary–adrenal (HPA) axis and sympathetic nervous system and culminates with elevated levels of glucocorticoids and catecholamines. Over time, the excessive accumulation of these substances can have downstream repercussions and contribute to subclinical or clinical conditions affecting the cardiovascular, metabolic, and immune systems. AL may provide a biosocial framework bridging SED and CP. First, individuals subjected to prolonged SEDs or deteriorating trajectories of such adversities are prone to trigger chronic stress responses and dysregulation across multiple physiological systems (33). In accordance with the theory, several years of empirical evidence consistently show that people who report more SED have higher AL levels (34, 35). Second, the pathology of CP is associated with the physiological dysregulation of systems related to AL (36–38). Specifically, the excessive activation of the HPA axis and sympathetic nervous system, accompanied by subsequent sustained pro-inflammatory states, is related to central sensitization and alterations in pain signaling pathways, resulting in the occurrence or maintenance of nociception (37, 39). Despite the compelling theory linking AL and CP, research investigating the correlation between these two factors, especially in terms of prospective population-based studies, remains scarce. Previous studies have indicated that CP was associated with the dysregulation of the biological stress response to chronic stress (38, 40). Cross-sectional studies based on population samples have discovered that elevated AL was associated with higher levels of CP severity and an increased risk of its occurrence (41, 42). However, previous studies did not incorporate HPA axis biomarkers in constructing AL (42), and the definition of CP used in other studies lacked time specificity (41). Moreover, early CP (30) and medication use (43) were not adjusted for.

Childhood has been recognized as a critical period in both the risk accumulation and social mobility models, and childhood SED could act as a moderator in the association between AL and CP. The critical period emphasizes that during childhood, which is considered a window of biological development, the experience of adversity can have long-lasting and irreversible effects on physiological functions (44). Numerous studies indicate that exposure to chronic stress during the critical period can alter the normal development of the stress response system (45) and disrupt the pain processing pathway (46), thereby exacerbating poor stress responses and amplifying the effects of stress on pain perception in adulthood. Although previous research on the critical period has emphasized the biological consequences of childhood disadvantage and emerging literature has revealed the long-term harmful effects of SED on AL and CP (23, 47), whether the biological long arm of childhood SED magnifies the impacts of chronic stress response on CP in adulthood remains unclear.

Overall, the present research aimed to examine three goals. The first goal was to test whether cumulative SED and SED trajectory would have a prospective association with future CP outcomes. Second, in preparation for mediation analysis, the present study aimed to examine whether SED and SED trajectory would be prospectively related to AL and examine whether AL would be associated with future CP outcomes. Also, the moderation effect of childhood SED on the association between AL and CP was tested. The third goal was to test whether AL would mediate the association between SED and CP, Furthermore, the study aimed to examine whether mediation analyses would be moderated by childhood SED.

## 2. Methods

### 2.1. Data

**Figure 1:**
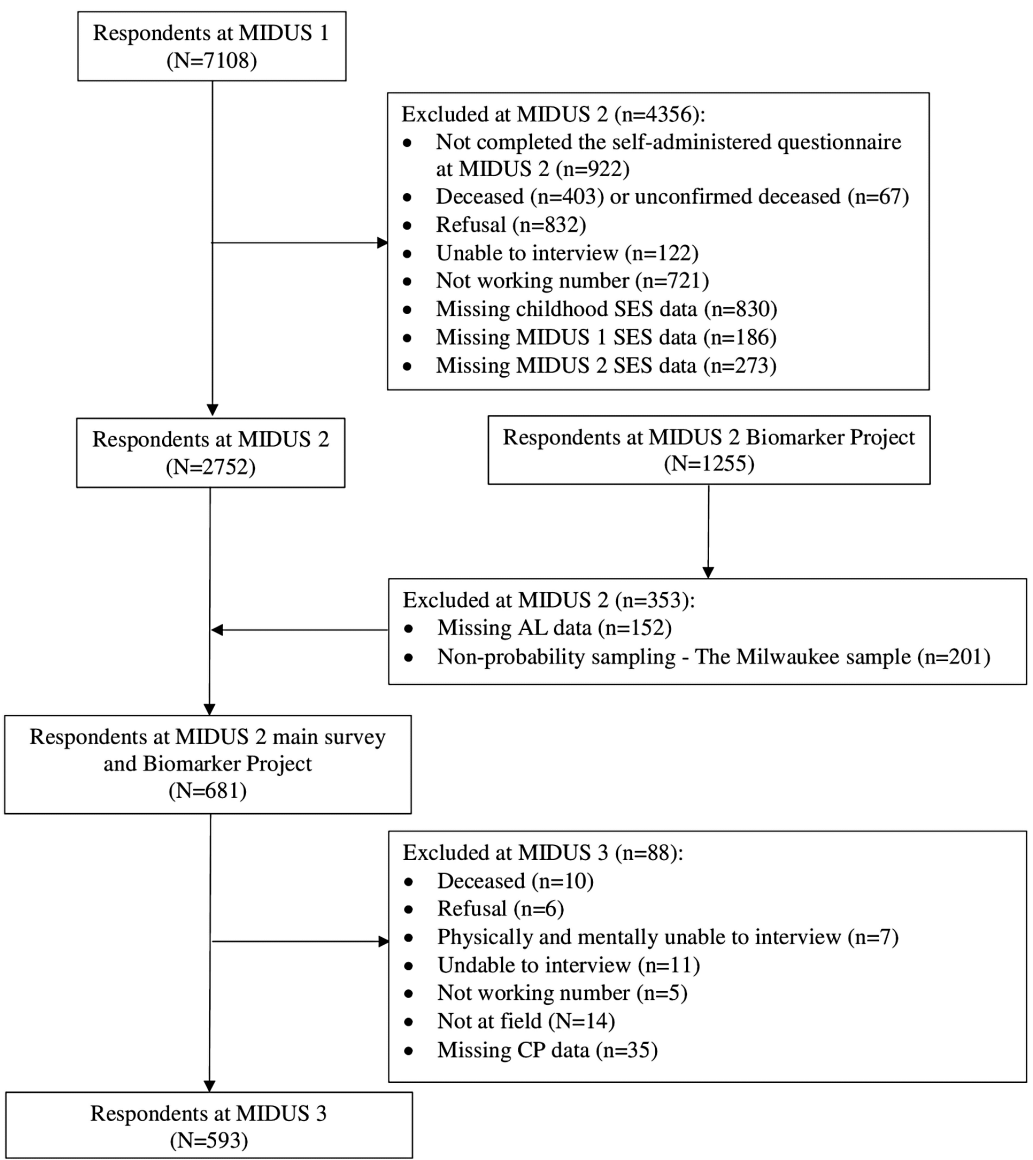
Flowchart for eligible sample.

This study used data from the Midlife in the United States (MIDUS) study, which spanned from 1995 to 2014, including three waves of main surveys and a biomarker project. The MIDUS is a national longitudinal study on individual social status, psychological profiles, and biological processes of aging, which started between 1995 and 1996 and followed 7,108 non-institutionalized Americans aged 25–74 years in the contiguous United States. The MIDUS 2 and MIDUS 3 main surveys followed the same group of respondents and collected data through phone interviews and self-administered questionnaires between 2004 and 2006, and 2013 and 2014, respectively. In addition, a total of 1,255 respondents participated in the MIDUS 2 biomarker project conducted from 2004 to 2009 with 201 non-probability samples of the Milwaukee project. The samples meeting the following criteria were incorporated into the final analysis (see Figure 1): (1) samples that completed the baseline survey and two MIDUS follow-up surveys and participated in the biomarker program and (2) samples that provided complete information on the major variables (SED, AL, and CP). The majority of MIDUS data are publicly available and can be accessed through the Inter-University Consortium of Political and Social Research (ICPSR) data repository (https://www.icpsr.umich.edu/web/ICPSR/series/203). However, because of the specificity of geographic and racial information involved in the MIDUS Milwaukee data, additional protection against the disclosure of personally identifying information is required. More information can be found at http://midus.wisc.edu/. With regard to the biomarker project, details are reported in description documents (48, 49).

### 2.2. Measures

#### 2.2.1. Dependent variable: chronic pain

CP is defined as pain that persists or recurs for more than 3 months (11). CP was measured using two indicators. The present study aimed to explore the prospective relationship between SEDs and CP; therefore, MIDUS 3 data regarding CP interference and the number of pain sites were utilized. The respondents were first asked, “Do you have CP, that is, do you have pain that persists beyond the time of normal healing and has lasted from anywhere from a few months to many years?” If they answered “yes,” they were then asked about CP interference. A pain interference index was generated by calculating the mean score from five questions of how much pain interfered with the respondents’ activity, mood, relations, sleep, and enjoyment, with scores ranging from 0 (no interference) to 10 (complete interference). The items for this index were selected based on the Pain Interference Subscale of the Brief Pain Inventory (50). Then, the CP interference index was further categorized as categorical variables into no pain (respondents without CP), low-interference pain (≤4), and high-interference pain (>4). In addition, if respondents reported experiencing CP, they were asked about the location of the pain across nine regions, including the head, neck, back, arms, legs, shoulders, hips, knees, and other sites. The pain sites were summed up into a count variable and then categorized into no pain, 0–2 sites, or 3 or more sites as a categorical variable. The threshold values for pain interference, the number of pain sites, and the calculation methods for the pain interference scale and the number of pain sites were determined based on established practices from previous studies (27, 43, 50).

#### 2.2.2. Allostatic load

AL biomarkers were collected from the MIDUS 2 biomarker project. The project collected 12-h urine samples, fasting blood samples, and data on nervous system function (48) from respondents during a one-day stay at the General Clinical Research Center of either UCLA, University of Wisconsin, or Georgetown University, depending on where the respondents live.

Building upon previous studies (20, 32), AL was constructed using 27 biomarkers from seven physiological systems (as shown in Table 1). The upper or lower quartiles were used to represent the high-risk threshold of biomarkers (51). Dehydroepiandrosterone sulfate (DHEA-S) and cortisol in their upper or lower 25th quartiles were considered high risk. When high-frequency heart rate variability (HFHRV), low-frequency heart rate variability (LFHRV), root mean square of successive differences (RMSSD), and standard deviation of heart beat–to–heart beat intervals (SDRR) fell within their lower 25th quartiles, they were considered high risk. Other biomarkers with values falling into their upper 25th quartile were assigned to the high-risk range. Biomarkers in their high-risk quartiles were coded as 1; otherwise, they were coded as 0. The AL index was then calculated by summing up the risk scores of the biomarkers (52), with a theoretical range of 0–27.

**Table 1:** High-risk cut-point values for AL biomarkers.

Algorithms	Simple high-risk quartile
Hypothalamic–pituitary–adrenal axis	
Hormone DHEA-S (µg/dl)	≤51 or ≥141
Urine cortisol (µg/g)	≤6.70 or ≥19
Sympathetic nervous system	
Urine epinephrine (µg/g)	≥2.46
Urine norepinephrine (µg/g)	≥32.96
Urine dopamine (µg/g)	≥182.96
Parasympathetic nervous system	
HFHRV	≤55.90
LFHRV	≤103.40
RMSSD	≤12.02
SDRR (ms)	≤23.27
Cardiovascular	
Resting heart rate (bpm)	≥79.80
Resting systolic blood pressure (SBP) (mmHg)	≥144
Resting diastolic blood pressure (DBP) (mmHg)	≥82
Metabolic—glucose	
Fasting glucose	≥105
Glycosylated hemoglobin (HbA1c) (%)	≥6.24
Homeostasis model of insulin resistance (HOMA-IR)	≥4.36
Metabolic—lipids	
Triglycerides (mg/dl)	≥156
Waist-to-hip ratio (WHR)	≥0.97
Body mass index (BMI)	≥33.03
Low-density lipoprotein (LDL) cholesterol (mg/dl)	≥127
High-density lipoprotein (HDL) cholesterol (mg/dl)	≤43
Inflammation	
C-reactive protein (CRP) (mg/L)	≥3.66
Interleukin-6 (IL6) (pg/ml)	≥1.23
Tumor necrosis factor-α (TNF-α) (pg/ml)	≥2.51
Fibrinogen (mg/dl)	≥399
Soluble endothelial leukocyte adhesion molecule-1 (sE-Selectin) (ng/ml)	≥51.88
Soluble intercellular adhesion molecule-1 (ICAM-1) (ng/ml)	≥335.19
Blood fasting insulin-like growth factor 1 (IGF1) (ng/ml)	≥157

#### 2.2.3. Socioeconomic disadvantages: accumulation and trajectories

The indicator selection and algorithm of SED were based on previous research (20, 53–55). SED was divided into three periods, namely, childhood, adulthood in MIDUS 1, and adulthood in MIDUS 2. In addition, each SED indicator was recoded into an index ranging from 0 to 2, where 0 represented no SED, 1 represented mild/moderate SED, and 2 represented severe SED. Childhood SED was retrospectively collected during the MIDUS 1 period, including the highest level of parental education (0 = bachelor's degree or more, 1 = high school/General Educational Development diploma (GED)/some college, or 2 = less than high school), whether a respondent was on welfare during childhood (0 = no, 2 = yes), and father's occupation (0 = managerial and professional specialty occupations/technical, sales, and administrative support occupations/service occupations; 1 = operators, fabricators, and laborers/farming, forestry, and fishing occupations; or 2 = precision production, craft, and repair occupations/experienced unemployed not classified by occupations).

SED during adulthood (MIDUS 1 and MIDUS 2) included the income-to-needs ratio adjusted for family size and year (0 = affluent/adequate income, 1 = low income, or 2 = poor/extreme poverty), education (0 = bachelor's degree or more, 1 = high school/GED/some college, or 2 = less than high school), rating of current financial situation (0 = best, 1 = medium, or 2 = worst), money to meet needs (0 = more than enough money, 1 = just enough money, or 2 = not enough money), difficulty to pay monthly bills (0 = not at all difficult, 1 = not very difficult/somewhat difficult, or 2 = very difficult), and occupation (0 = managerial and professional specialty occupations/technical, sales, and administrative support occupations/service occupations; 1 = operators, fabricators, and laborers/farming, forestry, and fishing occupations; or 2 = precision production, craft, and repair occupations/experienced unemployed not classified by occupations).

The cumulative indices of SED for childhood and adulthood were calculated by summing up the indicators within each period. In childhood, the index ranged from 0 to 8, while in adulthood (MIDUS 1 and MIDUS 2), it ranged from 0 to 12. The overall SED index, which represents the sum of the indices for all three periods, ranges from 0 to 32 (see Table 2).

**Table 2:** SED indicators used to generate the cumulative socioeconomic disadvantage index.

SED indicators	SED categories and values assigned		
	0 (least disadvantaged)	1 (medium)	2 (most disadvantaged)
Childhood SED			
Highest level of parental education (father's and mother's)	Bachelor's degree or more	High school/GED/some college	Less than high school
Welfare during childhood	No		Yes
Father's occupation (Census 1980 classification)^a^	(1) Managerial and professional specialty occupations (2) Technical, sales, and administrative support occupations (3) Service occupations	(1) Operators, fabricators, and laborers (2) Farming, forestry, and fishing occupations	(1) Precision production, craft, and repair occupations (2) Experienced unemployed not classified by occupations
Adulthood SED (MIDUS 1 and MIDUS 2)			
Income-to-needs ratio adjusted for family size and year	Affluent/adequate income	Low income	Poor/extreme poverty
Highest level of education	Bachelor's degree or more	High school/GED/some college	Less than high school
Rating of current financial situation	Best	Medium	Worst
Money to meet needs	More than enough money	Just enough money	Not enough money
Difficulty in paying monthly bills	Not at all difficult	Not very difficult/somewhat difficult	Very difficult
Occupation (Census 1980 classification for MIDUS 1, Census 1990 classification for MIDUS 2)^b^	(1) Managerial and professional specialty occupations (2) Technical, sales, and administrative support occupations (3) Service occupations	(1) Operators, fabricators, and laborers (2) Farming, forestry, and fishing occupations	(1) Precision production, craft, and repair occupations (2) Experienced unemployed not classified by occupations

^a^ Note that if a father never worked due to disability, addiction, or mental issues, they were classified as unemployed. If they did not work for other reasons, such as raising children at home, the mother's occupation represented the father's when the childhood occupation variable was constructed.

^b^ Note that if the current employment status of a respondent was unemployed, permanently disabled, never worked, or due to other reasons, they were classified as unemployed. If they were temporarily laid off, on maternity or sick leave, or retired, their occupation was represented by their last held position. If they were homemakers or part-time students, their spouse or partner's occupation was represented. Their father's occupation represents the occupation of a full-time student.

Latent class trajectory modeling (LCTM) was employed to identify groups of SED mobility. The method can simplify heterogeneous individuals in longitudinal changes into groups or clusters with similar trends (56, 57). LCTM was estimated using the R package “lcmm” (58). The trajectory group number was tested from 1 to 5, and the model performance was further improved by random effects (intercepts, slopes, or both), standardized SED index calculations (dividing the period disadvantage index by item count), and reduced skewness with square roots. The optimal trajectory clustering was determined by the lower values of the Akaike information criterion (AIC), the Bayesian information criterion (BIC), and sample size–adjusted BIC (SABIC); the assurance of a minimum of 5% of respondents per class; and the high entropy value (57, 59).

#### 2.2.4. Confounders

The selection of confounders was based on existing knowledge and research findings (20, 41, 43), which aims to screen for confounding effects that exist between SED and AL, SED and CP, and AL and CP, to minimize spurious associations (as shown in Supplementary Tables S1B–F).

With regard to the associations between childhood SED and CP/AL, gender, age at MIDUS 1, race/ethnicity, whether living with a smoker/alcoholic during childhood, whether living with biological parents during childhood, parental health, and emotional/physical abuse from mothers/fathers were adjusted for.

Concerning the links between adulthood SED at MIDUS 1/2 and CP/AL, gender, age at MIDUS 1/2, race/ethnicity, marital status at MIDUS 1/2, number of chronic conditions (60, 61) at MIDUS 1/2, support from family/friends at MIDUS 1/2, personal mastery at MIDUS 1/2, perceived constraint at MIDUS 1/2, smoking behavior at MIDUS 1/2, drinking behavior at MIDUS 1/2, and physical activity at MIDUS 1/2 were controlled for.

Regarding the associations between SED trajectories and CP/AL, we controlled for the confounders from childhood SED and CP/AL and from adulthood SED at MIDUS 1 and CP/AL.

With regard to the associations between AL and CP, we adjusted for gender, age at MIDUS 2, race/ethnicity, marital status at MIDUS 2, SED at MIDUS 2, emotional/physical abuse from mothers/fathers, perceived stress scale, number of chronic conditions at MIDUS 2 biomarker project (62), metabolic equivalent of task (MET), and medications including antihyperlipidemic agents, angiotensin-converting enzyme inhibitors, beta-adrenergic blocking agents, antihypertensive combinations, analgesics, anxiolytics sedatives and hypnotics, antiplatelet agents, antacids, sex hormones, thyroid hormones, antihistamines, antidepressants, analgesics, and opioid.

### 2.3. Statistical analyses

Multinomial logistic regression was used to estimate the odds ratio (OR) of CP interference and CP sites at MIDUS 3 in relation to cumulative SED (including life course cumulative SED and period-specific cumulative SED). This approach was chosen due to the categorical nature of the two pain measures. Similarly, multinomial logistic regression was used to test the association between SED trajectories and CP measures at MIDUS 3. All related confounders were adjusted for (for confounder information, see Section 2.2.4). The same approach was used to test the association between AL and CP. Finally, an interaction term of AL and childhood cumulative SED was included to examine the potential moderating effect of childhood SED on the association between AL and CP.

Negative binomial models were used to examine the association between cumulative SEDs at specific periods and across life course and AL, to control for relevant confounders. This method was used because AL was coded as a count variable. Finally, the associations between SED trajectories and AL were tested using negative binomial models.

Finally, causal mediation analyses were conducted using the R package “mediation” (63) to examine whether SEDs were indirectly associated with CP by the mediating effects of AL. We created 500 bootstrapped samples to estimate the indirect effect and its standard error. The counterfactual framework was introduced to mediation analysis, and the sequential ignorability assumption is a fundamental assumption in the causal mediation analysis (64). This assumption posits that the mediator must be conditionally independent of the effect, given the exposure and any other confounders that are measured or controlled for in the analysis. Compared with the traditional mediation analysis, causal mediation analysis shows more robustness to different limitations, including confounding between the exposure, mediator, and outcome variables. Due to the fact that the “mediation” package does not accommodate negative binomial regression as the method for analyzing mediating effects (SEDs-AL), nor does it support multinomial logistic regression as the method for examining direct effects (SEDs-CP), this study, in accordance with prior research, employed ordinary least squares linear regression for the mediating effect model (65) and employed logistic regression for the direct effect model after coding categorical CP measures into dummy variables (66). Also, moderated mediation analyses were conducted to examine whether childhood SEDs would modify the effect of AL on CP.

Missing data can lead to a biased estimation (67, 68). Multiple imputation (MI) using the R package “MICE” (69) was employed to address item non-response, based on the assumption of missing at random. It should be noted that MI can also be applied to handle missing completely at random (70). The missingness pattern of the data was assessed using Little's test (71), with the “misty” R package, to determine if it followed a systematic or random pattern. The result indicated that there was no evidence of systematic missingness (*P* = 0.999). Missing confounders were then imputed based on the specific distribution of each item, following the recommended approach (68). A total of 20 imputed datasets were generated, and the coefficients from all statistical models, including causal mediation analysis, were combined using Rubin's rules.

## 3. Results

### 3.1. Descriptive statistics

Supplementary Tables S1A–F present the characteristics of the analytic sample (*N* = 593). Similar distributions demonstrate the effectiveness of MI, and subsequent regression results are identical between the imputed data and the complete case data. A total of 381 respondents reported being free from pain, 144 individuals reported experiencing low-interference pain, and 68 individuals reported having high-interference pain. In addition, 133 individuals reported experiencing pain in fewer than two body regions, and 79 individuals reported experiencing pain in three or more body sites. Meanwhile, the AL index of the sample was moderate (with a mean of 6.7 and a median of 6). With regard to the number of SEDs, there was no significant difference between the three periods, with the number of SED events fluctuating around an average of approximately 2.9 events, while the average number of life course SED events was 8.8.

**Figure 2:**
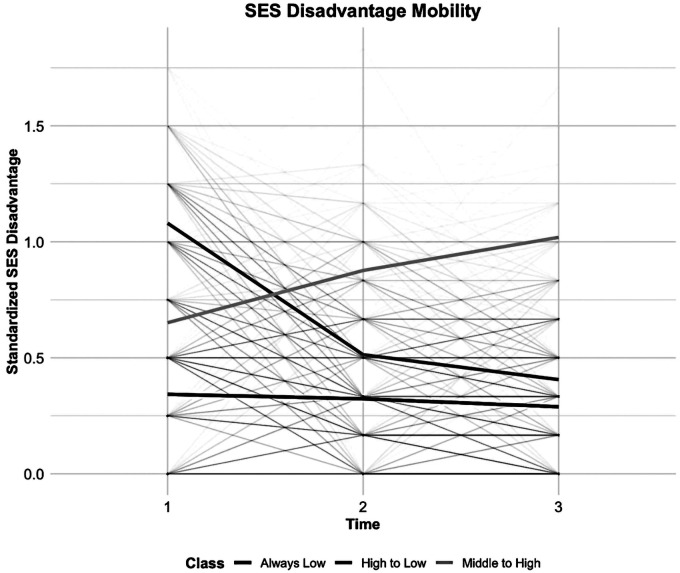
Group-based trajectories of socioeconomic disadvantages.

According to the model fit statistics (see Figure 2 and Table 3), the standardized SED index and random slope method of LCTM generated the best-fitting model for the classification. We identified three SED mobility groups, namely, stable low, early high to late low, and early medium to late high (lower scores indicating higher SED). Approximately 45.7% of participants had severe early SED that improved to mild late SED, 36.8% had stable low, and 17.5% had early moderate SED that worsened to late severe SED.

**Table 3:** Model fit of the latent class trajectory model.

Number of groups	loglik	conv	npm	AIC	BIC	SABIC	Entropy	%class1	%class2	%class3	%class4	%class5
*k* = 1	−3,651.75	1	6	7,315.50	7,341.81	7,322.76	1.00	100.00	NA	NA	NA	NA
*k* = 2	−3,630.92	1	9	7,279.85	7,319.31	7,290.74	0.68	82.97	17.03	NA	NA	NA
*k* = 3	−3,630.92	1	12	7,285.85	7,338.47	7,300.37	0.39	19.39	80.61	0.00	NA	NA
*k* = 4	−3,619.81	1	15	7,269.63	7,335.40	7,287.78	0.64	31.20	9.27	54.47	5.06	NA
*k* = 5	−3,615.78	1	18	7,267.56	7,346.49	7,289.35	0.57	28.50	9.44	4.38	32.04	25.63
Further improvement												
*k* = 3 (standardized index + random slope)	−281.70	1	12	587.40	640.02	601.93	0.54	17.54	36.76	45.70	NA	NA
*k* = 3 (squared root standardized index + random slope)	927.00	1	12	−1,829.99	−1,777.37	−1,815.46	0.68	32.72	17.88	49.41	NA	NA
*k* = 4 (random slope + intercept)	−9,080.86	1	15	18,191.73	18,257.50	18,209.88	0.24	0.00	25.46	74.54	0.00	NA
*k* = 4 (random intercept)	−9,109.59	1	13	18,245.18	18,302.18	18,260.91	0.81	48.57	24.45	15.51	11.47	NA
*k* = 4 (squared root index + random intercept)	−3,480.83	1	15	6,991.67	7,057.44	7,009.82	0.71	14.50	17.54	38.45	29.51	NA
*k* = 4 (squared root index + random intercept + slope)	−3,446.98	1	15	6,923.96	6,989.74	6,942.12	0.49	73.86	15.18	0.00	10.96	NA
*k* = 4 (standardized index + random slope)	−260.87	1	15	551.75	617.53	569.91	0.76	44.18	34.91	3.20	17.71	NA
*k* = 4 (squared root standardized index + random slope)	976.75	2	15	−1,923.50	−1,857.72	−1,905.34	0.84	0.00	75.89	10.12	14.00	NA

### 3.2. Regression results

#### 3.2.1. Associations between accumulation and trajectories of SEDs and CP

Table 4 presents the associations between period-specific cumulative SED, life course cumulative SED, and SED trajectories and the degree of CP interference and the number of pain sites at MIDUS 3. The results indicated that only cumulative SED at MIDUS 2 was associated with higher CP interference at MIDUS 3. The risk of reporting high pain interference increased by 15% (OR = 1.15, 95% CI: 1.01–1.32, *P* < 0.05) with each additional SED event experienced in MIDUS 2. However, no statistical evidence supported the lifetime accumulation of risk model.

**Table 4:** Results from the multinomial regression for the association between SEDs and CP interference and the number of pain sites.

No pain vs.	Multinomial regression			
	Pain interference		Number of pain locations	
	Low-interference pain	High-interference pain	0–2	3+
	Odds ratio (95%CI)		Odds ratio (95% CI)	
Main analysis				
Period-specific SED				
SEDs at childhood^a^	0.94 (0.83–1.07)	1.11 (0.94–1.31)	0.92 (0.81–1.04)	1.14 (0.97–1.34)
SEDs at MIDUS 1^b^	1.03 (0.94–1.14)	1.03 (0.90–1.18)	0.99 (0.89–1.10)	1.11 (0.98–1.26)
SEDs at MIDUS 2^c^	1.05 (0.95–1.16)	**1.15** (1.01–1.32)*	1.09 (0.99–1.21)	1.05 (0.92–1.20)
Lifetime disadvantages^c^				
Total SEDs	1.01 (0.97–1.06)	1.04 (0.97–1.12)	1.01 (0.96–1.06)	1.05 (0.98–1.12)
SED trajectory—class (*k* = 3, reference = always low)^a^,^c^				
Middle to high	1.46 (0.78–2.7)	**3.22** (1.35–7.71)**	1.37 (0.74–2.56)	**3.06** (1.28–7.32)*
High to low	0.89 (0.55–1.43)	1.69 (0.81–3.52)	0.85 (0.52–1.39)	1.77 (0.87–3.6)

^a^ Adjusted for gender, age at MIDUS 1, race/ethnicity, whether living with smoker/alcoholic during childhood, whether living with biological parents during childhood, parental health, and emotional/physical abuse from mothers/fathers.

^b^ Adjusted for gender, age at MIDUS 1, race/ethnicity, marital status at MIDUS 1, number of chronic conditions, support from family/friends at MIDUS 1, personal mastery at MIDUS 1, smoking behavior at MIDUS 1, drinking behavior at MIDUS 1, and activity at MIDUS 1.

^c^ Adjusted for gender, age at MIDUS 2, race/ethnicity, marital status at MIDUS 2, number of chronic conditions, support from family/friends at MIDUS 2, personal mastery at MIDUS 2, smoking behavior at MIDUS 2, drinking behavior at MIDUS 2, and activity at MIDUS 2.

* Significant at the 5% level.

** Significant at the 1% level.

*** Significant at the 0.1% level.

The bold values denote statistically significant results.

Furthermore, the results from the SED trajectory analysis showed that individuals who experienced a moderate number of SEDs in childhood and saw a worsening trend in adulthood reported higher pain interference (OR = 3.22, 95% CI: 1.35–7.71, *P* < 0.01) and three or more pain sites (OR = 3.06, 95% CI: 1.28–7.32, *P* < 0.05) at MIDUS 3, compared with those who continuously experience few SEDs. There was no difference in the likelihood of reporting CP conditions between individuals who transitioned to fewer SED events in later life from high SED conditions in childhood and those who consistently experienced low SED situations.

#### 3.2.2. Associations between SEDs and AL

Table 5 demonstrates that more SEDs at each period consistently correlate with increased prevalence of AL. Also, the prevalence of AL increased by 2% [incidence rate ratio (IRR) = 1.02, 95% CI: 1.01–1.03, *P* < 0.001] with each unit increment in cumulative SEDs.

**Table 5:** Results from the negative binomial regression for the association between SEDs and AL.

	Negative binomial regression
	AL index quartile-based
	IRR (95%CI)
Main analysis	
Period-specific SED	
SEDs at childhood^a^	**1.04** (1.01–1.07)**
SEDs at MIDUS 1^b^	**1.03** (1.01–1.06)**
SEDs at MIDUS 2^c^	**1.03** (1.01–1.05)**
Lifetime disadvantages^c^	
Total SEDs	**1.02** (1.01–1.03)***
SED trajectory—class (*k* = 3, reference = always low)^a^,^c^	
Middle to high	**1.19** (1.05–1.36)**
High to low	**1.11** (1.01–1.22)*

^a^ Adjusted for gender, age at MIDUS 1, race/ethnicity, whether living with smoker/alcoholic during childhood, whether living with biological parents during childhood, parental health, and emotional/physical abuse from mothers/fathers.

^b^ Adjusted for gender, age at MIDUS 1, race/ethnicity, marital status at MIDUS 1, number of chronic conditions, support from family/friends at MIDUS 1, personal mastery at MIDUS 1, smoking behavior at MIDUS 1, drinking behavior at MIDUS 1, and activity at MIDUS 1.

^c^ Adjusted for gender, age at MIDUS 2, race/ethnicity, marital status at MIDUS 2, number of chronic conditions, support from family/friends at MIDUS 2, personal mastery at MIDUS 2, smoking behavior at MIDUS 2, drinking behavior at MIDUS 2, and activity at MIDUS 2.

* Significant at the 5% level.

** Significant at the 1% level.

*** Significant at the 0.1% level.

The bold values denote statistically significant results.

Individuals who had a moderate number of SEDs in childhood and experienced continuous deterioration in SEDs throughout their lives had a 19% higher incidence rate of AL (IRR = 1.19, 95% CI: 1.05–1.36, *P* < 0.01), compared with those who consistently experienced few SEDs. Similarly, those who had a high number of SED early on and then experienced low disadvantage later in life had an 11% higher incidence rate (IRR = 1.11, 95% CI: 1.01–1.2, *P* < 0.05).

#### 3.2.3. The association between AL and CP and mediation analysis

The main analysis in Table 6 does not reveal a prospective relationship between AL and CP. However, subsequent moderation analysis found that childhood SED significantly strengthened the correlation between AL and CP in three or more pain sites. Specifically, with each additional high-risk AL biomarker and childhood SED, the risk of CP in no fewer than three sites increased by 19% (95% CI: 1.02–1.4, *P* < 0.05) and 69% (95% CI: 1.16–2.46, *P* < 0.01), respectively. Surprisingly, the impact of AL was weakened by increasing childhood SED events (OR = 0.95, 95% CI: 0.9–0.99, *P* < 0.05).

**Table 6:** Results from the multinomial regression for the association between AL and CP interference and the number of pain sites.

No pain vs.	Multinomial regression^a^			
	Pain interference		Number of pain locations	
	Low-interference pain	High-interference pain	0–2	3+
	Odds ratio (95%CI)		Odds ratio (95%CI)	
Main analysis				
AL index defined by high-risk quartiles	0.99 (0.93–1.06)	0.99 (0.89–1.09)	0.99 (0.92–1.06)	1 (0.92–1.09)
Interaction: SEDs at childhood* AL				
AL index defined by high-risk quartiles	1.02 (0.91–1.15)	1.1 (0.94–1.3)	0.98 (0.88–1.11)	**1.19** (1.02–1.4)*
SEDs at childhood	0.99 (0.77–1.27)	1.38 (0.93–2.05)	0.88 (0.68–1.15)	**1.69** (1.16–2.46)**
SEDs at childhood* AL	0.99 (0.96–1.02)	0.97 (0.92–1.01)	1 (0.97–1.04)	**0.95** (0.9–0.99)*

^a^ Adjusted for gender, age at MIDUS 2, race/ethnicity, marital status at MIDUS 2, SED at MIDUS 2, emotional/physical abuse from mothers/fathers, perceived stress scale, number of chronic conditions at MIDUS 2 biomarker project, MET, and medications including antihyperlipidemic agents, angiotensin-converting enzyme inhibitors, beta-adrenergic blocking agents, antihypertensive combinations, analgesics, anxiolytics sedatives and hypnotics, antiplatelet agents, antacids, sex hormones, thyroid hormones, antihistamines, antidepressants, analgesics, and opioid.

* Significant at the 5% level.

** Significant at the 1% level.

*** Significant at the 0.1% level.

The bold values denote statistically significant results.

Finally, mediation analyses, including the moderation mediation analysis of the interaction between childhood SED and AL, did not show significant mediating effects (see Supplementary Table S2B). The most probable pathway to achieve the mediating effect of AL was the moderation mediation analysis of childhood SED and three or more CP sites (Prop. mediated = 0.25, 95% CI: −0.12–1.72, *P* = 0.0608), but statistically significant evidence was not demonstrated.

## 4. Discussion

This study utilized the MIDUS national survey to examine the prospective associations between SED and CP. The results indicated a significant association between proximal SED and high-interference CP, but not between the whole life course accumulation of SED and future CP. Also, individuals with a worsening SED trajectory had a higher risk of high-interference pain and 3+ chronic pain sites. There is a positive correlation between cumulative SED and AL, and an improving trajectory of SED has a protective effect on AL. The association between AL and CP was initially not found significant, but after adding the interaction term of childhood SED and AL, the association became significant. Specifically, childhood SED may reduce the impact of AL on CP. Finally, no mediating effect of AL was found.

Previous studies have found that economic difficulties at age 43 and cumulative economic difficulties at ages 43 and 60–64 were associated with chronic widespread pain at age 68 (24). Similarly, another study found that occupational status at age 42 was associated with chronic widespread pain at age 45 (26). However, we did not find similar associations between cumulative SED and CP widespreadness. Compared with the studies mentioned, this study has several advantages that may contribute to the difference. We attempted to capture the multidimensional aspects of SED by integrating both subjective and objective SEDs rather than relying on a single socioeconomic indicator (72, 73). In addition, we considered various confounders to eliminate spurious associations. For example, disease diagnoses can reduce employment chances and working hours (74) and harm future health outcomes and social status (29). Therefore, chronic disease exacerbates SED and triggers CP conditions (30), leading to a false association. Furthermore, a different measure of CP widespreadness may contribute to the null association. Previous studies relied on the 1990 American College of Rheumatology standard (24, 26), while our measure utilized the 2016 revision criteria (75), which involved the use of the widespread pain index. However, both pain measurement methods utilized in the previous literature and in the MIDUS study were merely approximations of clinical pain assessment.

This study found that only MIDUS 2 SED was prospectively associated with high-interference CP at MIDUS 3; therefore, the results did not support the lifetime accumulation of risk model. This finding is consistent with previous research on life course SED and health outcome. A long-term cohort study of individuals who graduated from high school in Wisconsin in 1957 showed that only proximal socioeconomic status was associated with later mortality (22). The results of the present study corroborate previous research indicating that subsequent socioeconomic resources completely mediate the impacts of early SED on CP. Furthermore, in line with prior research, our results indicated that increased SEDs are related to elevated levels of CP interference (76–78). The recent accumulation of SED has exposed individuals to a high risk environment for CP and limited access to immediate resources for CP management. This could be one explanation for this association. Moreover, pain interference encompasses not only the intensity of pain but also the perceptions and beliefs of an individual about their pain (79). Individuals with recent socioeconomic advantages may hold more positive attitudes toward their health and possess greater self-efficacy in managing it, thereby contributing to a more favorable assessment of pain interference (80).

To our knowledge, our study is the first to use rigorously defined measures of CP to ascertain a prospective association of SED trajectories with CP within a community-dwelling sample. We used LCTM to standardize the trajectory phenotype of SED from heterogeneous data, providing a robust and reproducible tool for measuring SED trajectories (57), which supplements the limited literature on social mobility and CP. Our results support the social mobility model of CP development. The results indicated that individuals experiencing continuous worsening of SED over the years were more likely to have more CP sites and high-interference pain. A study found that a low socioeconomic trajectory was associated with high pain interference (81). However, SED trajectories were constructed using binary measures for three periods, which may result in an overclassified group number and groups with very few respondents. Another study refined the classification approach of unsecured debt-to-income ratio trajectories through a group-based trajectory model. They revealed that individuals who experienced trajectories of more unsecured debt were more susceptible to reporting joint problems and pain interference compared with those with minimal or no debt (82). However, both studies assessed pain experienced over the past 4 weeks rather than pain persisting for more than 3 months, resulting in the measure including trivial and recent pain. Our rigorous use of measures defined based on CP criteria eliminates much measurement error that previous studies with vague measures of CP duration have, thereby significantly enhancing our comprehension of the link between SED trajectories and CP.

Also, compared with those who remained in a prolonged state of advantage, no significant association was found between an improving SED trajectory and CP, even among individuals who experienced a highly disadvantaged childhood. One study found that an upward income trajectory can mitigate the adverse impacts of early-life disadvantages on physical health (19). Therefore, positive social mobility may enhance an individual’s access to flexible resources, which in turn enables them to adopt more proactive pain-coping strategies and cultivate healthy social relationships. As a result, this has the potential to alleviate the detrimental effects of early SED on the development of CP in the future. It could explain the null association between the trajectory characterized by high SED early in life transitioning to low SED later in life and CP.

This study may also be the first to explore the prospective association between AL and CP using population-based samples. Our results indicated that the relationship was initially statistically insignificant, but the association became significant when the interaction term of AL and childhood SED was added. On the one hand, as a critical period, childhood disadvantage can alter long-term pain processing, including sensitizing nociceptive pathways and lowering adulthood pain thresholds (46). In addition, early-life chronic stress can chronically change the function of the HPA axis, resulting in a pronociceptive effect (45, 46). As indicated by our results, childhood SED increases the risk of having AL, and AL was prospectively associated with having no less than three CP sites in the presence of the synergistic effect of SED and AL. On the other hand, we have discovered a counterintuitive phenomenon that childhood SED has a protective effect on at least three CP sites. One explanation is the development of resilience during childhood. Being resilient refers to the ability to effectively deal with the aftermath of risks, overcome the effects of traumatic events, and avoid harmful paths that frequently accompany risky circumstances (83). Childhood is a plastic period for developing resilience, and children raised in socioeconomically deprived environments may acquire coping skills and tactics that empower them to handle stress and adversity, potentially mitigating the harmful impact of disadvantage on their health (84–86), including CP (87, 88).

Only one community-dwelling study examined the mediating effect of AL on the cross-sectional association between SED and CP (42), and the findings indicate that the mediating effect was not significant. Similarly, when employing an introduced counterfactual framework in the mediation analysis, we did not find a significant prospective mediating effect of AL while controlling for additional confounders. The non-significant mediating effect may be related to the following reasons or limitations. First, different CP syndromes exhibit distinct underlying pathologies. The recent classification of CP (ICD-11) distinguishes between two broad categories (11), namely, chronic primary pain (also referred to as nociplastic pain) and chronic secondary pain (also categorized as nociceptive pain and neuropathic pain). Chronic primary pain is characterized by persistent or recurring pain in one or more anatomical regions lasting longer than 3 months (11). It is accompanied by significant emotional distress or functional interference, impacting daily activities and social engagement. This type of pain is not attributable to another specific CP condition (11) and is often associated with maladaptive stress responses (89). However, the CP measure in our study may have captured chronic secondary pain, which arises directly from underlying conditions such as cancer, stroke, diabetic neuropathy, and other related ailments (11). This is because our findings demonstrated a significant association between the chronic disease index and CP conditions (results not presented), indicating that diseases predominantly induced the pain. Future research can improve the analysis of AL as a mediator by using specific types of CP, such as migraine (36). Nevertheless, it is important to acknowledge that even for identical CP types, pain pathological traits may vary depending on the course, such as HPA dysfunction manifestation being associated with degrees of muscle pain distribution (38).

Second, this operationalization has limitations even if we measured AL using rigorously collected biomarkers that included the primary mediator system and calculated the AL index using classical additive methods. This additive method assumes that stressors have an equal impact on each high-risk biomarker or that each biomarker is equally crucial for outcomes. In addition, how the biomarkers of each score are combined is not clear. Alternative methods include latent class analysis and latent profile analysis. They can detect the dysregulation pattern of physiological systems driving AL (90). However, both latent class analysis and latent profile analysis come with their own set of challenges. One challenge is the exploratory nature of these analyses, which means that subjectivity often involves selecting the final model and qualitatively naming the clusters. We encourage future research to use advanced algorithms to narrow the gap between operationalization and theoretical implications.

There are also limitations to our sample. While the demographic and health traits of the biomarker project sample closely resemble those of the national survey sample (91), our longitudinal data containing biomarker project samples lack representation of ethnic minorities. Daily stress, such as discrimination, can amplify the CP inequality experienced by African Americans in the incidence of CP and medical practices (92). Therefore, future research should supplement the study of minority ethnic samples. In addition, childhood indicators were retrospectively collected. Some studies have validated the concordance of childhood socioeconomic indicators in the MIDUS study using sibling samples and a sample of twins, and the results indicated that the concordance of recall measures was generally high (93). However, prospective childhood indicators are still recommended in future research due to retrospective biases.

## 5. Conclusion

This study revealed the prospective associations between recent SED and future CP reports despite limitations. Also, the present study supports the social mobility model of CP. Furthermore, childhood, as a critical developmental period, provides advantages in developing resilience to adversity through the experience of SED. However, this resilience comes at a cost, as it interacts with AL and increases the risk of multisite pain in adulthood. Overall, increasing opportunities for positive social mobility and reducing childhood and recent SED may be key to mitigating inequalities in CP. Future research might investigate SED and CP connections by refining CP and AL measurements or scrutinizing other sociobiological processes.

## Data Availability

The data backing the conclusions of this study can be found openly at the Inter-university Consortium for Political and Social Research. MIDUS 1: 10.3886/ICPSR02760.v19. MIDUS 2: 10.3886/ICPSR04652.v8. MIDUS 2 Biomarker Project: 10.3886/ICPSR29282.v10. MIDUS 3: 10.3886/ICPSR36346.v7.
