# Supplementary material for: The mediating effect of allostatic load on the association between life course socioeconomic disadvantage and chronic pain: a prospective finding from the National Survey of Midlife Development in the United States
Source: Front Pain Res (Lausanne). 2023 Jul 13;4:1213750. doi: 10.3389/fpain.2023.1213750 (PMC10374263; doi:10.3389/fpain.2023.1213750)
Supplement: Supplementary file 1 [file Table1.docx]

Supplementary Material

# Supplementary Tables 1a-f: Characteristics of analytical samples (N=593)

Supplementary Table 1a Characteristics of analytical sample (N = 593)

|  | Observed | | | |  | Imputed | | | |
| --- | --- | --- | --- | --- | --- | --- | --- | --- | --- |
| **Variable** | **Mean / N** | **SD / Proportion** | **Median** | **Missing %** |  | **Mean / N** | **SD / Proportion** | **Median** | **Test** |
| **Dependent variable** |  |  |  |  |  |  |  |  |  |
| Pain interference at MIDUS 3 | 593 |  |  | 0.00% |  |  |  |  | X^2^=0 |
| No pain (Ref) | 381 | 64.20% |  |  |  |  | 64.20% |  |  |
| Low interference pain | 144 | 24.30% |  |  |  |  | 24.30% |  |  |
| High interference pain | 68 | 11.50% |  |  |  |  | 11.50% |  |  |
| The number of pain locations at MIDUS 3 | 593 |  |  | 0.00% |  |  |  |  | X^2^=0 |
| No pain (Ref) | 381 | 64.20% |  |  |  |  | 64.20% |  |  |
| 0-2 | 133 | 22.40% |  |  |  |  | 22.40% |  |  |
| 3+ | 79 | 13.30% |  |  |  |  | 13.30% |  |  |
| **Mediator - AL** |  |  |  |  |  |  |  |  |  |
| AL index defined by high-risk quartiles | 6.7 | 3.69 | 6 | 0.00% |  | 6.7 | 3.69 | 6 | F=0 |
| **SEDs** |  |  |  |  |  |  |  |  |  |
| SEDs during childhood | 2.9 | 1.74 | 3 | 0.00% |  | 2.9 | 1.73 | 3 | F=0 |
| SEDs at MIDUS 1 | 3 | 2.15 | 3 | 0.00% |  | 3 | 2.15 | 3 | F=0 |
| SEDs at MIDUS 2 | 2.8 | 2.20 | 3 | 0.00% |  | 2.8 | 2.20 | 3 | F=0 |
| Lifetime SED index | 8.8 | 4.62 | 9 | 0.00% |  | 8.8 | 4.61 | 9 | F=0 |
| SEDs trajectory class | 593 |  |  | 0.00% |  |  |  |  | X^2^=0 |
| Always low (Ref) | 218 | 36.80% |  |  |  |  | 36.80% |  |  |
| Middle to high | 104 | 17.50% |  |  |  |  | 17.50% |  |  |
| High to low | 271 | 45.70% |  |  |  |  | 45.70% |  |  |

Supplementary Table 1b Characteristics of analytical sample (N = 593)

|  | Observed | | | |  | Imputed | | | |
| --- | --- | --- | --- | --- | --- | --- | --- | --- | --- |
| **Variable** | **Mean / N** | **SD / Proportion** | **Median** | **Missing %** |  | **Mean / N** | **SD / Proportion** | **Median** | **Test** |
| **Sociodemographic confounders** |  |  |  |  |  |  |  |  |  |
| Gender | 593 |  |  | 0.00% |  |  |  |  | X^2^=0 |
| Male (Ref) | 281 | 47.40% |  |  |  |  | 47.40% |  |  |
| Female | 312 | 52.60% |  |  |  |  | 52.60% |  |  |
| Age at MIDUS 1 | 44.6 | 10.79 | 44 | 0.00% |  | 44.6 | 10.78 | 44 | F=0 |
| Age at MIDUS 2 | 53.6 | 10.78 | 53 | 0.00% |  | 53.6 | 10.77 | 53 | F=0 |
| Race/Ethnicity | 593 |  |  | 0.00% |  |  |  |  | X^2^=0 |
| White (Ref) | 561 | 94.60% |  |  |  |  | 94.60% |  |  |
| non-White | 32 | 5.40% |  |  |  |  | 5.40% |  |  |
| Marital status at MIDUS 1 | 593 |  |  | 0.00% |  |  |  |  | X^2^=0 |
| Married (Ref) | 429 | 72.30% |  |  |  |  | 72.30% |  |  |
| Divorced&Separated&Widowed | 86 | 14.50% |  |  |  |  | 14.50% |  |  |
| Never married | 78 | 13.20% |  |  |  |  | 13.20% |  |  |
| Marital status at MIDUS 2 | 592 |  |  | 0.17% |  |  |  |  | X^2^=0.003 |
| Married (Ref) | 431 | 72.80% |  |  |  |  | 72.70% |  |  |
| Divorced&Separated&Widowed | 100 | 16.90% |  |  |  |  | 17% |  |  |

Supplementary Table 1c Characteristics of analytical sample (N = 593)

|  | Observed | | | |  | Imputed | | | |
| --- | --- | --- | --- | --- | --- | --- | --- | --- | --- |
| **Variable** | **Mean / N** | **SD / Proportion** | **Median** | **Missing %** |  | **Mean / N** | **SD / Proportion** | **Median** | **Test** |
| **Childhood confounders** |  |  |  |  |  |  |  |  |  |
| Whether lived with smoker during childhood | 593 |  |  | 0.00% |  |  |  |  | X^2^=0 |
| Yes (Ref) | 187 | 31.50% |  |  |  |  | 31.50% |  |  |
| No | 406 | 68.50% |  |  |  |  | 68.50% |  |  |
| Whether lived with alcoholic during childhood | 592 |  |  | 0.17% |  |  |  |  | X^2^=0 |
| Yes (Ref) | 119 | 20.10% |  |  |  |  | 20.20% |  |  |
| No | 473 | 79.90% |  |  |  |  | 79.80% |  |  |
| Whether lived with biological parents | 593 |  |  | 0.00% |  |  |  |  | X^2^=0 |
| Yes (Ref) | 510 | 86% |  |  |  |  | 86% |  |  |
| No | 83 | 14% |  |  |  |  | 14% |  |  |
| Mothers past health | 588 |  |  | 0.84% |  |  |  |  | X^2^=0.005 |
| Excellent (Ref) | 116 | 19.70% |  |  |  |  | 19.70% |  |  |
| Deceased | 15 | 2.60% |  |  |  |  | 2.60% |  |  |
| Fair | 63 | 10.70% |  |  |  |  | 10.80% |  |  |
| Good | 151 | 25.70% |  |  |  |  | 25.60% |  |  |
| Poor | 29 | 4.90% |  |  |  |  | 4.90% |  |  |
| Very good | 214 | 36.40% |  |  |  |  | 36.30% |  |  |
| Fathers past health | 579 |  |  | 2.36% |  |  |  |  | X^2^=0.055 |
| Excellent (Ref) | 129 | 22.30% |  |  |  |  | 22.20% |  |  |
| Deceased | 20 | 3.50% |  |  |  |  | 3.60% |  |  |
| Fair | 56 | 9.70% |  |  |  |  | 9.80% |  |  |
| Good | 144 | 24.90% |  |  |  |  | 24.80% |  |  |
| Poor | 27 | 4.70% |  |  |  |  | 4.80% |  |  |
| Very good | 203 | 35.10% |  |  |  |  | 34.80% |  |  |
| Emotional abuse - mother | 579 |  |  | 2.36% |  |  |  |  | X^2^=0.083 |
| Often (Ref) | 35 | 6% |  |  |  |  | 6.20% |  |  |
| Never | 286 | 49.40% |  |  |  |  | 48.80% |  |  |
| Rarely | 171 | 29.50% |  |  |  |  | 29.70% |  |  |
| Sometimes | 87 | 15% |  |  |  |  | 15.30% |  |  |
| Emotional abuse - father | 582 |  |  | 1.85% |  |  |  |  | X^2^=0.049 |
| Often (Ref) | 48 | 8.20% |  |  |  |  | 8.50% |  |  |
| Never | 239 | 41.10% |  |  |  |  | 40.90% |  |  |
| Rarely | 177 | 30.40% |  |  |  |  | 30.40% |  |  |
| Sometimes | 118 | 20.30% |  |  |  |  | 20.20% |  |  |
| Physical abuse - mother | 586 |  |  | 1.18% |  |  |  |  | X^2^=0.097 |
| Often (Ref) | 24 | 4.10% |  |  |  |  | 4.20% |  |  |
| Never | 328 | 56% |  |  |  |  | 55.60% |  |  |
| Rarely | 171 | 29.20% |  |  |  |  | 29.10% |  |  |
| Sometimes | 63 | 10.80% |  |  |  |  | 11.10% |  |  |
| Physical abuse - father | 588 |  |  | 0.84% |  |  |  |  | X^2^=0.034 |
| Often (Ref) | 25 | 4.30% |  |  |  |  | 4.40% |  |  |
| Never | 311 | 52.90% |  |  |  |  | 52.80% |  |  |
| Rarely | 176 | 29.90% |  |  |  |  | 29.80% |  |  |
| Sometimes | 76 | 12.90% |  |  |  |  | 13.10% |  |  |

Supplementary Table 1d Characteristics of analytical sample (N = 593)

|  | Observed | | | |  | Imputed | | | |
| --- | --- | --- | --- | --- | --- | --- | --- | --- | --- |
| **Variable** | **Mean / N** | **SD / Proportion** | **Median** | **Missing %** |  | **Mean / N** | **SD / Proportion** | **Median** | **Test** |
| **MIDUS 1 confounders** |  |  |  |  |  |  |  |  |  |
| Number of Chronic Conditions (12 months) at MIDUS 1 | 2.1 | 2.12 | 1 | 0.00% |  | 2.1 | 2.12 | 1 | F=0 |
| Support from family at MIDUS 1 | 3.5 | 0.58 | 3.75 | 0.30% |  | 3.5 | 0.58 | 3.75 | F=0.001 |
| Support from friends at MIDUS 1 | 3.3 | 0.62 | 3.25 | 0.20% |  | 3.3 | 0.62 | 3.25 | F=0 |
| Personal mastery at MIDUS 1 | 5.9 | 0.96 | 6 | 0.20% |  | 5.9 | 0.96 | 6 | F=0 |
| Perceived constraints at MIDUS 1 | 2.4 | 1.06 | 2.23 | 0.20% |  | 2.4 | 1.06 | 2.23 | F=0 |
| Smoking at MIDUS 1 | 593 |  |  | 0.00% |  |  |  |  | X^2^=0 |
| Current smoker (Ref) | 72 | 12.10% |  |  |  |  | 12.10% |  |  |
| Ex-smoker | 348 | 58.70% |  |  |  |  | 58.70% |  |  |
| Non-smoker | 173 | 29.20% |  |  |  |  | 29.20% |  |  |
| Drinking at MIDUS 1 | 593 |  |  | 0.00% |  |  |  |  | X^2^=0 |
| Moderate + Drinker (Ref) | 358 | 60.40% |  |  |  |  | 60.40% |  |  |
| Light Drinker | 198 | 33.40% |  |  |  |  | 33.40% |  |  |
| Non-Drinker or rarley Drink | 37 | 6.20% |  |  |  |  | 6.20% |  |  |
| Physical activity index at MIDUS 1 | 4.9 | 1.02 | 5.25 | 0.30% |  | 4.9 | 1.02 | 5.25 | F=0 |

Supplementary Table 1e Characteristics of analytical sample (N = 593)

|  | Observed | | | |  | Imputed | | | |
| --- | --- | --- | --- | --- | --- | --- | --- | --- | --- |
| **Variable** | **Mean / N** | **SD / Proportion** | **Median** | **Missing %** |  | **Mean / N** | **SD / Proportion** | **Median** | **Test** |
| **MIDUS 2 confounders** |  |  |  |  |  |  |  |  |  |
| Number of Chronic Conditions (12 months) at MIDUS 2 | 2 | 2.12 | 2 | 0.00% |  | 2 | 2.12 | 2 | F=0 |
| Support from family at MIDUS 2 | 3.5 | 0.57 | 3.75 | 0.20% |  | 3.5 | 0.57 | 3.75 | F=0 |
| Support from friends at MIDUS 2 | 3.4 | 0.61 | 3.5 | 0.70% |  | 3.4 | 0.61 | 3.5 | F=0 |
| Personal mastery at MIDUS 2 | 5.8 | 0.99 | 6 | 0.20% |  | 5.8 | 0.99 | 6 | F=0 |
| Perceived constraints at MIDUS 2 | 2.3 | 1.04 | 2.13 | 0.20% |  | 2.3 | 1.04 | 2.13 | F=0 |
| Smoking at MIDUS 2 | 593 |  |  | 0.00% |  |  |  |  | X^2^=0 |
| Current smoker (Ref) | 58 | 9.80% |  |  |  |  | 9.80% |  |  |
| Ex-smoker | 367 | 61.90% |  |  |  |  | 61.90% |  |  |
| Non-smoker | 168 | 28.30% |  |  |  |  | 28.30% |  |  |
| Drinking at MIDUS 2 | 593 |  |  | 0.00% |  |  |  |  | X^2^=0 |
| Moderate + Drinker (Ref) | 216 | 36.40% |  |  |  |  | 36.40% |  |  |
| Light Drinker | 175 | 29.50% |  |  |  |  | 29.50% |  |  |
| Non-Drinker or rarley Drink | 202 | 34.10% |  |  |  |  | 34.10% |  |  |
| Physical activity index at MIDUS 2 | 30.3 | 10.22 | 30.08 | 4.60% |  | 30.20 | 10.23 | 30 | F=0.047 |
| CP at MIDUS 2 | 584 |  |  | 1.52% |  |  |  |  | X^2^=0 |
| No (Ref) | 407 | 69.70% |  |  |  |  | 69.70% |  |  |
| Yes | 177 | 30.30% |  |  |  |  | 30.30% |  |  |

Supplementary Table 1f Characteristics of analytical sample (N = 593)

|  | Observed | | | |  | Imputed | | | |
| --- | --- | --- | --- | --- | --- | --- | --- | --- | --- |
| **Variable** | **Mean / N** | **SD / Proportion** | **Median** | **Missing %** |  | **Mean / N** | **SD / Proportion** | **Median** | **Test** |
| **MIDUS 2 biomarker project confounders** |  |  |  |  |  |  |  |  |  |
| Total number of Symptoms and Chronic Conditions - biomarker project | 3.7 | 2.75 | 3 | 0.00% |  | 3.7 | 2.75 | 3 | F=0 |
| Total number of Metabolic Equivalent of Task (MET) minutes per week | 590 |  |  | 0.51% |  |  |  |  | X^2^=0.001 |
| 500-1000 (Ref) | 115 | 19.50% |  |  |  |  | 19.50% |  |  |
| Greater than 1000 | 249 | 42.20% |  |  |  |  | 42.20% |  |  |
| Less than 500 | 226 | 38.30% |  |  |  |  | 38.30% |  |  |
| Antihyperlipidemic agents | 592 |  |  | 0.17% |  |  |  |  | X^2^=0 |
| Yes (Ref) | 174 | 29.40% |  |  |  |  | 29.50% |  |  |
| No | 418 | 70.60% |  |  |  |  | 70.50% |  |  |
| Angiotensin converting enzyme inhibitors | 592 |  |  | 0.17% |  |  |  |  | X^2^=0 |
| Yes (Ref) | 45 | 7.60% |  |  |  |  | 7.60% |  |  |
| No | 547 | 92.40% |  |  |  |  | 92.40% |  |  |
| Beta adrenergic blocking agents | 592 |  |  | 0.17% |  |  |  |  | X^2^=0 |
| Yes (Ref) | 72 | 12.20% |  |  |  |  | 12.20% |  |  |
| No | 520 | 87.80% |  |  |  |  | 87.80% |  |  |
| Antihypertensive combinations | 592 |  |  | 0.17% |  |  |  |  | X^2^=0 |
| Yes (Ref) | 53 | 9% |  |  |  |  | 8.90% |  |  |
| No | 539 | 91% |  |  |  |  | 91.10% |  |  |
| Analgesics | 592 |  |  | 0.17% |  |  |  |  | X^2^=0 |
| Yes (Ref) | 299 | 50.50% |  |  |  |  | 50.50% |  |  |
| No | 293 | 49.50% |  |  |  |  | 49.50% |  |  |
| Anxiolytics sedatives and hypnotics | 592 |  |  | 0.17% |  |  |  |  | X^2^=0 |
| Yes (Ref) | 74 | 12.50% |  |  |  |  | 12.50% |  |  |
| No | 518 | 87.50% |  |  |  |  | 87.50% |  |  |
| Antiplatelet agents | 592 |  |  | 0.17% |  |  |  |  | X^2^=0 |
| Yes (Ref) | 175 | 29.60% |  |  |  |  | 29.50% |  |  |
| No | 417 | 70.40% |  |  |  |  | 70.50% |  |  |
| Antacids | 592 |  |  | 0.17% |  |  |  |  | X^2^=0 |
| Yes (Ref) | 76 | 12.80% |  |  |  |  | 12.80% |  |  |
| No | 516 | 87.20% |  |  |  |  | 87.20% |  |  |
| Sex hormones | 592 |  |  | 0.17% |  |  |  |  | X^2^=0 |
| Yes (Ref) | 68 | 11.50% |  |  |  |  | 11.50% |  |  |
| No | 524 | 88.50% |  |  |  |  | 88.50% |  |  |
| Thyroid hormones | 592 |  |  | 0.17% |  |  |  |  | X^2^=0 |
| Yes (Ref) | 65 | 11% |  |  |  |  | 11% |  |  |
| No | 527 | 89% |  |  |  |  | 89% |  |  |
| Antihistamines | 592 |  |  | 0.17% |  |  |  |  | X^2^=0 |
| Yes (Ref) | 55 | 9.30% |  |  |  |  | 9.30% |  |  |
| No | 537 | 90.70% |  |  |  |  | 90.70% |  |  |
| Antidepressants | 592 |  |  | 0.17% |  |  |  |  | X^2^=0 |
| Yes (Ref) | 81 | 13.70% |  |  |  |  | 13.70% |  |  |
| No | 511 | 86.30% |  |  |  |  | 86.30% |  |  |
| Analgesic, opioid | 592 |  |  | 0.17% |  |  |  |  | X^2^=0 |
| Yes (Ref) | 28 | 4.70% |  |  |  |  | 4.70% |  |  |
| No | 564 | 95.30% |  |  |  |  | 95.30% |  |  |
| Perceived Stress Scale | 21.3 | 5.905 | 21 | 0.20% |  | 21.3 | 5.913 | 21 | F=0.005 |
