# Supplementary material for: The mediating effect of allostatic load on the association between life course socioeconomic disadvantage and chronic pain: a prospective finding from the National Survey of Midlife Development in the United States
Source: Front Pain Res (Lausanne). 2023 Jul 13;4:1213750. doi: 10.3389/fpain.2023.1213750 (PMC10374263; doi:10.3389/fpain.2023.1213750)
Supplement: Supplementary file 2 [file Table2.docx]

Supplementary Material

# Supplementary Tables 2: Results for longitudinal mediation analysis

Supplementary Table 2a SED trajectory-AL-CP

| **Main mediation** | |  |  |  |  |  |  |
| --- | --- | --- | --- | --- | --- | --- | --- |
| **Outcome:** | **High Interference Pain** | |  |  |  |  |  |
|  | **Social Trajectory - class (k=3, reference=2)** | | | | | | |
|  | **Middle to high** | | |  | **High to low** | | |
| **lm-glm** |  | Estimate | p-value |  |  | Estimate | p-value |
| **1** | ACME (control) | 0.01 (0, 0.02) | 0.273 |  | ACME (control) | 0 (0, 0.01) | 0.3104 |
| **2** | ACME (treated) | 0.01 (-0.01, 0.03) | 0.2726 |  | ACME (treated) | 0 (0, 0.01) | 0.3104 |
| **3** | ADE (control) | 0.09 (0, 0.21) | 0.0596 |  | ADE (control) | 0.04 (-0.03, 0.1) | 0.293 |
| **4** | ADE (treated) | 0.1 (0, 0.21) | 0.0596 |  | ADE (treated) | 0.04 (-0.03, 0.11) | 0.293 |
| **5** | Total Effect | **0.1 (0, 0.21)** | **0.0406** |  | Total Effect | 0.04 (-0.03, 0.11) | 0.2548 |
| **6** | Prop. Mediated (control) | 0.05 (-0.1, 0.57) | 0.3104 |  | Prop. Mediated (control) | 0.03 (-0.72, 0.85) | 0.5052 |
| **7** | Prop. Mediated (treated) | 0.07 (-0.12, 0.6) | 0.3096 |  | Prop. Mediated (treated) | 0.04 (-0.67, 0.86) | 0.5052 |
| **8** | ACME (average) | 0.01 (0, 0.02) | 0.2728 |  | ACME (average) | 0 (0, 0.01) | 0.3104 |
| **9** | ADE (average) | 0.09 (0, 0.21) | 0.0596 |  | ADE (average) | 0.04 (-0.03, 0.11) | 0.293 |
| **10** | Prop. Mediated (average) | 0.06 (-0.11, 0.59) | 0.31 |  | Prop. Mediated (average) | 0.04 (-0.7, 0.86) | 0.5052 |
| **Outcome:** | **Low Interference Pain** | |  |  |  |  |  |
|  | **Social Trajectory - class (k=3, reference=2)** | | | | | | |
|  | **Middle to high** | | |  | **High to low** | | |
| **lm-glm** |  | Estimate | p-value |  |  | Estimate | p-value |
| **1** | ACME (control) | 0 (-0.02, 0.01) | 0.6996 |  | ACME (control) | 0 (-0.01, 0.01) | 0.7044 |
| **2** | ACME (treated) | 0 (-0.02, 0.01) | 0.6996 |  | ACME (treated) | 0 (-0.01, 0.01) | 0.7044 |
| **3** | ADE (control) | 0.03 (-0.07, 0.15) | 0.5816 |  | ADE (control) | -0.03 (-0.11, 0.06) | 0.5334 |
| **4** | ADE (treated) | 0.03 (-0.07, 0.15) | 0.5816 |  | ADE (treated) | -0.03 (-0.11, 0.05) | 0.5334 |
| **5** | Total Effect | 0.03 (-0.07, 0.14) | 0.6066 |  | Total Effect | -0.03 (-0.11, 0.05) | 0.5146 |
| **6** | Prop. Mediated (control) | -0.02 (-1.42, 1.45) | 0.8426 |  | Prop. Mediated (control) | 0.01 (-0.81, 0.8) | 0.8578 |
| **7** | Prop. Mediated (treated) | -0.02 (-1.45, 1.46) | 0.8426 |  | Prop. Mediated (treated) | 0.01 (-0.82, 0.8) | 0.8578 |
| **8** | ACME (average) | 0 (-0.02, 0.01) | 0.6996 |  | ACME (average) | 0 (-0.01, 0.01) | 0.7044 |
| **9** | ADE (average) | 0.03 (-0.07, 0.15) | 0.5816 |  | ADE (average) | -0.03 (-0.11, 0.06) | 0.5334 |
| **10** | Prop. Mediated (average) | -0.02 (-1.44, 1.45) | 0.8426 |  | Prop. Mediated (average) | 0.01 (-0.81, 0.8) | 0.8578 |
| **Outcome:** | **3+ Pain** |  |  |  |  |  |  |
|  | **Social Trajectory - class (k=3, reference=2)** | | | | | | |
|  | **Middle to high** | | |  | **High to low** | | |
| **lm-glm** |  | Estimate | p-value |  |  | Estimate | p-value |
| **1** | ACME (control) | 0 (-0.01, 0.01) | 0.6806 |  | ACME (control) | 0 (0, 0.01) | 0.695 |
| **2** | ACME (treated) | 0 (-0.01, 0.02) | 0.6806 |  | ACME (treated) | 0 (-0.01, 0.01) | 0.695 |
| **3** | ADE (control) | **0.12 (0.01, 0.24)** | **0.0246** |  | ADE (control) | 0.06 (-0.01, 0.13) | 0.1194 |
| **4** | ADE (treated) | **0.12 (0.02, 0.24)** | **0.0246** |  | ADE (treated) | 0.06 (-0.01, 0.13) | 0.1194 |
| **5** | Total Effect | **0.12 (0.02, 0.24)** | **0.0204** |  | Total Effect | 0.06 (-0.01, 0.13) | 0.105 |
| **6** | Prop. Mediated (control) | 0.01 (-0.09, 0.24) | 0.6906 |  | Prop. Mediated (control) | 0.01 (-0.24, 0.4) | 0.7536 |
| **7** | Prop. Mediated (treated) | 0.02 (-0.12, 0.27) | 0.6906 |  | Prop. Mediated (treated) | 0.01 (-0.24, 0.42) | 0.7536 |
| **8** | ACME (average) | 0 (-0.01, 0.02) | 0.6806 |  | ACME (average) | 0 (0, 0.01) | 0.695 |
| **9** | ADE (average) | **0.12 (0.01, 0.24)** | **0.0246** |  | ADE (average) | 0.06 (-0.01, 0.13) | 0.1194 |
| **10** | Prop. Mediated (average) | 0.02 (-0.1, 0.26) | 0.6906 |  | Prop. Mediated (average) | 0.01 (-0.24, 0.41) | 0.7536 |
| **Outcome:** | **0-2 Pain** |  |  |  |  |  |  |
|  | **Social Trajectory - class (k=3, reference=2)** | | | | | | |
|  | **Middle to high** | | |  | **High to low** | | |
| **lm-glm** |  | Estimate | p-value |  |  | Estimate | p-value |
| **1** | ACME (control) | 0 (-0.01, 0.01) | 0.900 |  | ACME (control) | 0 (-0.01, 0.01) | 0.912 |
| **2** | ACME (treated) | 0 (-0.01, 0.01) | 0.900 |  | ACME (treated) | 0 (-0.01, 0.01) | 0.912 |
| **3** | ADE (control) | 0.02 (-0.08, 0.13) | 0.716 |  | ADE (control) | -0.03 (-0.11, 0.04) | 0.401 |
| **4** | ADE (treated) | 0.02 (-0.08, 0.13) | 0.716 |  | ADE (treated) | -0.03 (-0.11, 0.04) | 0.401 |
| **5** | Total Effect | 0.02 (-0.08, 0.13) | 0.722 |  | Total Effect | -0.03 (-0.11, 0.04) | 0.399 |
| **6** | Prop. Mediated (control) | -0.01 (-1.32, 1.29) | 0.951 |  | Prop. Mediated (control) | 0 (-0.72, 0.71) | 0.915 |
| **7** | Prop. Mediated (treated) | -0.01 (-1.34, 1.29) | 0.951 |  | Prop. Mediated (treated) | 0 (-0.72, 0.71) | 0.915 |
| **8** | ACME (average) | 0 (-0.01, 0.01) | 0.900 |  | ACME (average) | 0 (-0.01, 0.01) | 0.912 |
| **9** | ADE (average) | 0.02 (-0.08, 0.13) | 0.716 |  | ADE (average) | -0.03 (-0.11, 0.04) | 0.401 |
| **10** | Prop. Mediated (average) | -0.01 (-1.33, 1.29) | 0.951 |  | Prop. Mediated (average) | 0 (-0.72, 0.71) | 0.915 |
|  |  |  |  |  |  |  |  |
| **Moderated mediation** | |  |  |  |  |  |  |
| **Outcome:** | **High Interference Pain** | |  |  |  |  |  |
|  | **Social Trajectory - class (k=3, reference=2)** | | | | | | |
|  | **Middle to high** | | |  | **High to low** | | |
| **lm-glm** |  | Estimate | p-value |  |  | Estimate | p-value |
|  | ACME (control) | 0 (0, 0.02) | 0.279 |  | ACME (control) | 0 (0, 0.01) | 0.316 |
|  | ACME (treated) | 0.01 (-0.01, 0.02) | 0.275 |  | ACME (treated) | 0 (0, 0.01) | 0.319 |
|  | ADE (control) | 0.09 (-0.01, 0.22) | 0.098 |  | ADE (control) | 0.04 (-0.07, 0.15) | 0.472 |
|  | ADE (treated) | 0.1 (-0.01, 0.23) | 0.098 |  | ADE (treated) | 0.04 (-0.07, 0.15) | 0.472 |
|  | Total Effect | 0.1 (-0.01, 0.23) | 0.071 |  | Total Effect | 0.04 (-0.07, 0.15) | 0.444 |
|  | Prop. Mediated (control) | 0.04 (-0.21, 0.6) | 0.336 |  | Prop. Mediated (control) | 0.02 (-0.61, 0.71) | 0.623 |
|  | Prop. Mediated (treated) | 0.06 (-0.19, 0.62) | 0.332 |  | Prop. Mediated (treated) | 0.03 (-0.57, 0.72) | 0.623 |
|  | ACME (average) | 0.01 (0, 0.02) | 0.276 |  | ACME (average) | 0 (0, 0.01) | 0.318 |
|  | ADE (average) | 0.1 (-0.01, 0.23) | 0.098 |  | ADE (average) | 0.04 (-0.07, 0.15) | 0.472 |
|  | Prop. Mediated (average) | 0.05 (-0.2, 0.61) | 0.333 |  | Prop. Mediated (average) | 0.02 (-0.59, 0.71) | 0.624 |
| **Outcome:** | **Low Interference Pain** | |  |  |  |  |  |
|  | **Social Trajectory - class (k=3, reference=2)** | | | | | | |
|  | **Middle to high** | | |  | **High to low** | | |
| **lm-glm** |  | Estimate | p-value |  |  | Estimate | p-value |
|  | ACME (control) | 0 (-0.02, 0.01) | 0.732 |  | ACME (control) | 0 (-0.01, 0.01) | 0.740 |
|  | ACME (treated) | 0 (-0.02, 0.01) | 0.731 |  | ACME (treated) | 0 (-0.01, 0.01) | 0.739 |
|  | ADE (control) | 0.05 (-0.06, 0.17) | 0.438 |  | ADE (control) | 0.01 (-0.11, 0.13) | 0.866 |
|  | ADE (treated) | 0.05 (-0.06, 0.17) | 0.438 |  | ADE (treated) | 0.01 (-0.11, 0.13) | 0.866 |
|  | Total Effect | 0.05 (-0.06, 0.17) | 0.457 |  | Total Effect | 0.01 (-0.11, 0.13) | 0.886 |
|  | Prop. Mediated (control) | -0.02 (-1.33, 1.1) | 0.831 |  | Prop. Mediated (control) | 0 (-0.73, 0.77) | 0.956 |
|  | Prop. Mediated (treated) | -0.02 (-1.36, 1.1) | 0.831 |  | Prop. Mediated (treated) | 0 (-0.75, 0.76) | 0.956 |
|  | ACME (average) | 0 (-0.02, 0.01) | 0.731 |  | ACME (average) | 0 (-0.01, 0.01) | 0.738 |
|  | ADE (average) | 0.05 (-0.06, 0.17) | 0.438 |  | ADE (average) | 0.01 (-0.11, 0.13) | 0.866 |
|  | Prop. Mediated (average) | -0.02 (-1.35, 1.1) | 0.831 |  | Prop. Mediated (average) | 0 (-0.74, 0.76) | 0.956 |
| **Outcome:** | **3+ Pain** |  |  |  |  |  |  |
|  | **Social Trajectory - class (k=3, reference=2)** | | | | | | |
|  | **Middle to high** | | |  | **High to low** | | |
| **lm-glm** |  | Estimate | p-value |  |  | Estimate | p-value |
|  | ACME (control) | 0 (-0.01, 0.01) | 0.938 |  | ACME (control) | 0 (-0.01, 0.01) | 0.916 |
|  | ACME (treated) | 0 (-0.01, 0.02) | 0.887 |  | ACME (treated) | 0 (-0.01, 0.01) | 0.915 |
|  | ADE (control) | 0.1 (0, 0.22) | 0.060 |  | ADE (control) | 0.02 (-0.07, 0.12) | 0.635 |
|  | ADE (treated) | 0.1 (0, 0.22) | 0.060 |  | ADE (treated) | 0.02 (-0.07, 0.12) | 0.635 |
|  | Total Effect | 0.1 (0, 0.22) | 0.054 |  | Total Effect | 0.02 (-0.07, 0.12) | 0.636 |
|  | Prop. Mediated (control) | 0 (-0.24, 0.26) | 0.932 |  | Prop. Mediated (control) | 0 (-0.6, 0.61) | 0.898 |
|  | Prop. Mediated (treated) | 0.01 (-0.27, 0.3) | 0.902 |  | Prop. Mediated (treated) | 0.01 (-0.59, 0.62) | 0.858 |
|  | ACME (average) | 0 (-0.01, 0.01) | 0.929 |  | ACME (average) | 0 (-0.01, 0.01) | 0.915 |
|  | ADE (average) | 0.1 (0, 0.22) | 0.060 |  | ADE (average) | 0.02 (-0.07, 0.12) | 0.635 |
|  | Prop. Mediated (average) | 0 (-0.26, 0.28) | 0.939 |  | Prop. Mediated (average) | 0.01 (-0.59, 0.61) | 0.879 |
| **Outcome:** | **0-2 Pain** |  |  |  |  |  |  |
|  | **Social Trajectory - class (k=3, reference=2)** | | | | | | |
|  | **Middle to high** | | |  | **High to low** | | |
| **lm-glm** |  | Estimate | p-value |  |  | Estimate | p-value |
|  | ACME (control) | 0 (-0.01, 0.01) | 0.943 |  | ACME (control) | 0 (-0.01, 0.01) | 0.944 |
|  | ACME (treated) | 0 (-0.01, 0.01) | 0.938 |  | ACME (treated) | 0 (-0.01, 0.01) | 0.948 |
|  | ADE (control) | 0.05 (-0.06, 0.17) | 0.379 |  | ADE (control) | 0.04 (-0.08, 0.15) | 0.569 |
|  | ADE (treated) | 0.05 (-0.06, 0.17) | 0.379 |  | ADE (treated) | 0.04 (-0.08, 0.16) | 0.569 |
|  | Total Effect | 0.05 (-0.06, 0.17) | 0.380 |  | Total Effect | 0.04 (-0.08, 0.16) | 0.565 |
|  | Prop. Mediated (control) | 0 (-0.86, 0.85) | 0.959 |  | Prop. Mediated (control) | 0 (-0.47, 0.45) | 0.954 |
|  | Prop. Mediated (treated) | 0 (-0.86, 0.85) | 0.957 |  | Prop. Mediated (treated) | 0 (-0.47, 0.45) | 0.962 |
|  | ACME (average) | 0 (-0.01, 0.01) | 0.941 |  | ACME (average) | 0 (-0.01, 0.01) | 0.948 |
|  | ADE (average) | 0.05 (-0.06, 0.17) | 0.379 |  | ADE (average) | 0.04 (-0.08, 0.15) | 0.569 |
|  | Prop. Mediated (average) | 0 (-0.86, 0.85) | 0.958 |  | Prop. Mediated (average) | 0 (-0.47, 0.45) | 0.958 |

Supplementary Table 2b SED at childhood-AL-CP

| **Main mediation** |  |  |
| --- | --- | --- |
| **Outcome:** | **High Interference Pain** |  |
| **Individual SES disadvantages (concurrent disadvantage.)** | | |
| **SEDs at childhood** |  |  |
|  | Estimate | p-value |
| ACME (control) | 0 (0, 0) | 0.1006 |
| ACME (treated) | 0 (0, 0) | 0.1006 |
| ADE (control) | 0.01 (-0.01, 0.02) | 0.4038 |
| ADE (treated) | 0.01 (-0.01, 0.02) | 0.4038 |
| Total Effect | 0.01 (-0.01, 0.02) | 0.3166 |
| Prop. Mediated (control) | 0.1 (-1.48, 1.67) | 0.386 |
| Prop. Mediated (treated) | 0.1 (-1.45, 1.66) | 0.386 |
| ACME (average) | 0 (0, 0) | 0.1006 |
| ADE (average) | 0.01 (-0.01, 0.02) | 0.4038 |
| Prop. Mediated (average) | 0.1 (-1.46, 1.67) | 0.386 |
| **Outcome:** | **Low Interference Pain** |  |
| **Individual SES disadvantages (concurrent disadvantage.)** | | |
| **SEDs at childhood** |  |  |
|  | Estimate | p-value |
| ACME (control) | 0 (0, 0) | 0.9626 |
| ACME (treated) | 0 (0, 0) | 0.9626 |
| ADE (control) | -0.01 (-0.04, 0.01) | 0.3388 |
| ADE (treated) | -0.01 (-0.04, 0.01) | 0.3388 |
| Total Effect | -0.01 (-0.04, 0.01) | 0.3328 |
| Prop. Mediated (control) | 0 (-0.94, 0.83) | 0.9514 |
| Prop. Mediated (treated) | 0 (-0.94, 0.83) | 0.9514 |
| ACME (average) | 0 (0, 0) | 0.9626 |
| ADE (average) | -0.01 (-0.04, 0.01) | 0.3388 |
| Prop. Mediated (average) | 0 (-0.94, 0.83) | 0.9514 |
| **Outcome:** | **3+ Pain** |  |
| **Individual SES disadvantages (concurrent disadvantage.)** | | |
| **SEDs at childhood** |  |  |
|  | Estimate | p-value |
| ACME (control) | 0 (0, 0) | 0.8974 |
| ACME (treated) | 0 (0, 0) | 0.8974 |
| ADE (control) | -0.02 (-0.05, 0) | 0.134 |
| ADE (treated) | -0.02 (-0.05, 0) | 0.134 |
| Total Effect | -0.02 (-0.05, 0) | 0.139 |
| Prop. Mediated (control) | -0.01 (-0.52, 0.41) | 0.9196 |
| Prop. Mediated (treated) | -0.01 (-0.51, 0.41) | 0.9196 |
| ACME (average) | 0 (0, 0) | 0.8974 |
| ADE (average) | -0.02 (-0.05, 0) | 0.134 |
| Prop. Mediated (average) | -0.01 (-0.52, 0.41) | 0.9196 |
| **Outcome:** | **0-2 Pain** |  |
| **Individual SES disadvantages (concurrent disadvantage.)** | | |
| **SEDs at childhood** |  |  |
|  | Estimate | p-value |
| ACME (control) | 0 (0, 0) | 0.207 |
| ACME (treated) | 0 (0, 0) | 0.207 |
| ADE (control) | 0.01 (0, 0.02) | 0.087 |
| ADE (treated) | 0.01 (0, 0.02) | 0.087 |
| Total Effect | 0.01 (0, 0.02) | 0.054 |
| Prop. Mediated (control) | 0.06 (-0.17, 0.77) | 0.256 |
| Prop. Mediated (treated) | 0.07 (-0.17, 0.77) | 0.256 |
| ACME (average) | 0 (0, 0) | 0.207 |
| ADE (average) | 0.01 (0, 0.02) | 0.087 |
| Prop. Mediated (average) | 0.07 (-0.17, 0.77) | 0.256 |
|  |  |  |
| **Moderated mediation** |  |  |
| **Outcome:** | **High Interference Pain** |  |
| **Individual SES disadvantages (concurrent disadvantage.)** | | |
| **SEDs at childhood** |  |  |
|  | Estimate | p-value |
| ACME (control) | 0 (0, 0.01) | 0.172 |
| ACME (treated) | 0 (0, 0.01) | 0.115 |
| ADE (control) | 0.01 (-0.01, 0.02) | 0.275 |
| ADE (treated) | 0.01 (-0.01, 0.02) | 0.289 |
| Total Effect | 0.01 (-0.01, 0.02) | 0.174 |
| Prop. Mediated (control) | 0.17 (-1.61, 2.21) | 0.295 |
| Prop. Mediated (treated) | 0.17 (-1.3, 1.88) | 0.250 |
| ACME (average) | 0 (0, 0.01) | 0.143 |
| ADE (average) | 0.01 (-0.01, 0.02) | 0.280 |
| Prop. Mediated (average) | 0.17 (-1.44, 2.03) | 0.270 |
| **Outcome:** | **Low Interference Pain** |  |
| **Individual SES disadvantages (concurrent disadvantage.)** | | |
| **SEDs at childhood** |  |  |
|  | Estimate | p-value |
| ACME (control) | 0 (0, 0.01) | 0.777 |
| ACME (treated) | 0 (0, 0) | 0.827 |
| ADE (control) | -0.01 (-0.03, 0.01) | 0.424 |
| ADE (treated) | -0.01 (-0.03, 0.01) | 0.406 |
| Total Effect | -0.01 (-0.03, 0.01) | 0.459 |
| Prop. Mediated (control) | 0 (-2.02, 2.12) | 0.959 |
| Prop. Mediated (treated) | 0 (-1.52, 1.56) | 0.958 |
| ACME (average) | 0 (0, 0.01) | 0.799 |
| ADE (average) | -0.01 (-0.03, 0.01) | 0.414 |
| Prop. Mediated (average) | 0 (-1.75, 1.85) | 0.958 |
| **Outcome:** | **3+ Pain** |  |
| **Individual SES disadvantages (concurrent disadvantage.)** | | |
| **SEDs at childhood** |  |  |
|  | Estimate | p-value |
| ACME (control) | **0 (0, 0.01)** | **0.016** |
| ACME (treated) | **0 (0, 0.01)** | **0.017** |
| ADE (control) | 0.01 (0, 0.02) | 0.134 |
| ADE (treated) | 0.01 (-0.01, 0.02) | 0.179 |
| Total Effect | **0.01 (0, 0.02)** | **0.048** |
| Prop. Mediated (control) | 0.27 (-0.14, 1.96) | 0.061 |
| Prop. Mediated (treated) | 0.23 (-0.1, 1.48) | 0.062 |
| ACME (average) | **0 (0, 0.01)** | **0.016** |
| ADE (average) | 0.01 (0, 0.02) | 0.157 |
| Prop. Mediated (average) | 0.25 (-0.12, 1.72) | 0.061 |
| **Outcome:** | **0-2 Pain** |  |
| **Individual SES disadvantages (concurrent disadvantage.)** | | |
| **SEDs at childhood** |  |  |
|  | Estimate | p-value |
| ACME (control) | 0 (-0.01, 0) | 0.332 |
| ACME (treated) | 0 (-0.01, 0) | 0.460 |
| ADE (control) | -0.02 (-0.05, 0) | 0.116 |
| ADE (treated) | -0.02 (-0.05, 0) | 0.124 |
| Total Effect | -0.02 (-0.05, 0) | 0.103 |
| Prop. Mediated (control) | 0.1 (-0.42, 0.75) | 0.367 |
| Prop. Mediated (treated) | 0.05 (-0.34, 0.54) | 0.481 |
| ACME (average) | 0 (-0.01, 0) | 0.380 |
| ADE (average) | -0.02 (-0.05, 0) | 0.119 |
| Prop. Mediated (average) | 0.08 (-0.37, 0.63) | 0.409 |

Supplementary Table 2c SED at MIDUS 1-AL-CP

| **Main mediation** | |  |
| --- | --- | --- |
|  |  |  |
| **Individual SES disadvantages (concurrent disadvantage.)** | | |
| **SEDs at MIDUS 1** | |  |
|  | Estimate | p-value |
| ACME (control) | 0 (0, 0) | 0.0644 |
| ACME (treated) | 0 (0, 0) | 0.0644 |
| ADE (control) | 0 (-0.01, 0.01) | 0.8816 |
| ADE (treated) | 0 (-0.01, 0.01) | 0.8816 |
| Total Effect | 0 (-0.01, 0.01) | 0.683 |
| Prop. Mediated (control) | 0.15 (-4.07, 4.02) | 0.699 |
| Prop. Mediated (treated) | 0.16 (-4.01, 3.99) | 0.699 |
| ACME (average) | 0 (0, 0) | 0.0644 |
| ADE (average) | 0 (-0.01, 0.01) | 0.8816 |
| Prop. Mediated (average) | 0.15 (-4.04, 4.01) | 0.699 |
|  |  |  |
| **Individual SES disadvantages (concurrent disadvantage.)** | | |
| **SEDs at MIDUS 1** | |  |
|  | Estimate | p-value |
| ACME (control) | 0 (0, 0) | 0.7604 |
| ACME (treated) | 0 (0, 0) | 0.7604 |
| ADE (control) | 0 (-0.01, 0.02) | 0.6014 |
| ADE (treated) | 0 (-0.01, 0.02) | 0.6014 |
| Total Effect | 0 (-0.01, 0.02) | 0.6298 |
| Prop. Mediated (control) | -0.02 (-1.55, 1.48) | 0.8882 |
| Prop. Mediated (treated) | -0.02 (-1.56, 1.48) | 0.8882 |
| ACME (average) | 0 (0, 0) | 0.7604 |
| ADE (average) | 0 (-0.01, 0.02) | 0.6014 |
| Prop. Mediated (average) | -0.02 (-1.55, 1.48) | 0.8882 |
|  |  |  |
| **Individual SES disadvantages (concurrent disadvantage.)** | | |
| **SEDs at MIDUS 1** | |  |
|  | Estimate | p-value |
| ACME (control) | 0 (0, 0) | 0.6826 |
| ACME (treated) | 0 (0, 0) | 0.6826 |
| ADE (control) | -0.01 (-0.03, 0.01) | 0.474 |
| ADE (treated) | -0.01 (-0.03, 0.01) | 0.474 |
| Total Effect | -0.01 (-0.03, 0.01) | 0.501 |
| Prop. Mediated (control) | -0.02 (-1.4, 1.38) | 0.862 |
| Prop. Mediated (treated) | -0.02 (-1.4, 1.38) | 0.862 |
| ACME (average) | 0 (0, 0) | 0.6826 |
| ADE (average) | -0.01 (-0.03, 0.01) | 0.474 |
| Prop. Mediated (average) | -0.02 (-1.4, 1.38) | 0.862 |
|  |  |  |
| **Individual SES disadvantages (concurrent disadvantage.)** | | |
| **SEDs at MIDUS 1** | |  |
|  | Estimate | p-value |
| ACME (control) | 0 (0, 0) | 0.389 |
| ACME (treated) | 0 (0, 0) | 0.389 |
| ADE (control) | 0.01 (0, 0.02) | 0.112 |
| ADE (treated) | 0.01 (0, 0.02) | 0.112 |
| Total Effect | 0.01 (0, 0.02) | 0.082 |
| Prop. Mediated (control) | 0.06 (-0.32, 0.76) | 0.438 |
| Prop. Mediated (treated) | 0.06 (-0.32, 0.76) | 0.438 |
| ACME (average) | 0 (0, 0) | 0.389 |
| ADE (average) | 0.01 (0, 0.02) | 0.112 |
| Prop. Mediated (average) | 0.06 (-0.32, 0.76) | 0.438 |
|  |  |  |
| **Moderated mediation** | |  |
|  |  |  |
| **Individual SES disadvantages (concurrent disadvantage.)** | | |
| **SEDs at MIDUS 1** | |  |
|  | Estimate | p-value |
| ACME (control) | 0 (0, 0) | 0.087 |
| ACME (treated) | 0 (0, 0) | 0.087 |
| ADE (control) | 0 (-0.01, 0.01) | 0.937 |
| ADE (treated) | 0 (-0.01, 0.01) | 0.937 |
| Total Effect | 0 (-0.01, 0.01) | 0.862 |
| Prop. Mediated (control) | 0.08 (-3.49, 3.87) | 0.859 |
| Prop. Mediated (treated) | 0.09 (-3.44, 3.83) | 0.859 |
| ACME (average) | 0 (0, 0) | 0.087 |
| ADE (average) | 0 (-0.01, 0.01) | 0.937 |
| Prop. Mediated (average) | 0.08 (-3.46, 3.85) | 0.859 |
|  |  |  |
| **Individual SES disadvantages (concurrent disadvantage.)** | | |
| **SEDs at MIDUS 1** | |  |
|  | Estimate | p-value |
| ACME (control) | 0 (0, 0) | 0.821 |
| ACME (treated) | 0 (0, 0) | 0.822 |
| ADE (control) | 0.01 (-0.01, 0.02) | 0.492 |
| ADE (treated) | 0.01 (-0.01, 0.02) | 0.492 |
| Total Effect | 0.01 (-0.01, 0.02) | 0.499 |
| Prop. Mediated (control) | -0.01 (-1.16, 1.21) | 0.910 |
| Prop. Mediated (treated) | -0.01 (-1.16, 1.21) | 0.912 |
| ACME (average) | 0 (0, 0) | 0.822 |
| ADE (average) | 0.01 (-0.01, 0.02) | 0.492 |
| Prop. Mediated (average) | -0.01 (-1.16, 1.21) | 0.911 |
|  |  |  |
| **Individual SES disadvantages (concurrent disadvantage.)** | | |
| **SEDs at MIDUS 1** | |  |
|  | Estimate | p-value |
| ACME (control) | 0 (0, 0) | 0.660 |
| ACME (treated) | 0 (0, 0) | 0.652 |
| ADE (control) | 0.01 (0, 0.02) | 0.210 |
| ADE (treated) | 0.01 (0, 0.02) | 0.210 |
| Total Effect | 0.01 (0, 0.02) | 0.178 |
| Prop. Mediated (control) | 0.03 (-0.73, 0.93) | 0.739 |
| Prop. Mediated (treated) | 0.03 (-0.72, 0.93) | 0.730 |
| ACME (average) | 0 (0, 0) | 0.656 |
| ADE (average) | 0.01 (0, 0.02) | 0.210 |
| Prop. Mediated (average) | 0.03 (-0.72, 0.93) | 0.735 |
|  |  |  |
| **Individual SES disadvantages (concurrent disadvantage.)** | | |
| **SEDs at MIDUS 1** | |  |
|  | Estimate | p-value |
| ACME (control) | 0 (0, 0) | 0.647 |
| ACME (treated) | 0 (0, 0) | 0.647 |
| ADE (control) | 0 (-0.02, 0.01) | 0.662 |
| ADE (treated) | 0 (-0.02, 0.01) | 0.662 |
| Total Effect | 0 (-0.02, 0.01) | 0.707 |
| Prop. Mediated (control) | -0.01 (-1.73, 1.76) | 0.913 |
| Prop. Mediated (treated) | -0.01 (-1.73, 1.76) | 0.914 |
| ACME (average) | 0 (0, 0) | 0.647 |
| ADE (average) | 0 (-0.02, 0.01) | 0.662 |
| Prop. Mediated (average) | -0.01 (-1.73, 1.76) | 0.913 |

Supplementary Table 2d SED at MIDUS 2-AL-CP

| **Main mediation** | |  |
| --- | --- | --- |
|  |  |  |
| **Individual SES disadvantages (concurrent disadvantage.)** | | |
| **SEDs at MIDUS 2** | |  |
|  | Estimate | p-value |
| ACME (control) | 0 (0, 0) | 0.482 |
| ACME (treated) | 0 (0, 0) | 0.482 |
| ADE (control) | 0.01 (0, 0.02) | 0.0766 |
| ADE (treated) | 0.01 (0, 0.02) | 0.0766 |
| Total Effect | 0.01 (0, 0.02) | 0.0576 |
| Prop. Mediated (control) | 0.04 (-0.23, 0.48) | 0.5108 |
| Prop. Mediated (treated) | 0.04 (-0.24, 0.49) | 0.5108 |
| ACME (average) | 0 (0, 0) | 0.482 |
| ADE (average) | 0.01 (0, 0.02) | 0.0766 |
| Prop. Mediated (average) | 0.04 (-0.23, 0.48) | 0.5108 |
|  |  |  |
| **Individual SES disadvantages (concurrent disadvantage.)** | | |
| **SEDs at MIDUS 2** | |  |
|  | Estimate | p-value |
| ACME (control) | 0 (0, 0) | 0.7752 |
| ACME (treated) | 0 (0, 0) | 0.7752 |
| ADE (control) | 0 (-0.02, 0.02) | 0.7798 |
| ADE (treated) | 0 (-0.02, 0.02) | 0.7798 |
| Total Effect | 0 (-0.02, 0.02) | 0.8082 |
| Prop. Mediated (control) | -0.01 (-1.52, 1.59) | 0.9482 |
| Prop. Mediated (treated) | -0.01 (-1.52, 1.59) | 0.9482 |
| ACME (average) | 0 (0, 0) | 0.7752 |
| ADE (average) | 0 (-0.02, 0.02) | 0.7798 |
| Prop. Mediated (average) | -0.01 (-1.52, 1.59) | 0.9482 |
|  |  |  |
| **Individual SES disadvantages (concurrent disadvantage.)** | | |
| **SEDs at MIDUS 2** | |  |
|  | Estimate | p-value |
| ACME (control) | 0 (0, 0) | 0.6604 |
| ACME (treated) | 0 (0, 0) | 0.6604 |
| ADE (control) | 0 (-0.01, 0.01) | 0.8116 |
| ADE (treated) | 0 (-0.01, 0.01) | 0.8116 |
| Total Effect | 0 (-0.01, 0.01) | 0.7646 |
| Prop. Mediated (control) | 0.02 (-1.64, 1.61) | 0.8802 |
| Prop. Mediated (treated) | 0.02 (-1.63, 1.61) | 0.8802 |
| ACME (average) | 0 (0, 0) | 0.6604 |
| ADE (average) | 0 (-0.01, 0.01) | 0.8116 |
| Prop. Mediated (average) | 0.02 (-1.64, 1.61) | 0.8802 |
|  |  |  |
| **Individual SES disadvantages (concurrent disadvantage.)** | | |
| **SEDs at MIDUS 2** | |  |
|  | Estimate | p-value |
| ACME (control) | 0 (0, 0) | 0.934 |
| ACME (treated) | 0 (0, 0) | 0.934 |
| ADE (control) | 0.01 (0, 0.02) | 0.158 |
| ADE (treated) | 0.01 (0, 0.02) | 0.158 |
| Total Effect | 0.01 (0, 0.02) | 0.152 |
| Prop. Mediated (control) | -0.01 (-0.57, 0.61) | 0.929 |
| Prop. Mediated (treated) | -0.01 (-0.57, 0.61) | 0.929 |
| ACME (average) | 0 (0, 0) | 0.934 |
| ADE (average) | 0.01 (0, 0.02) | 0.158 |
| Prop. Mediated (average) | -0.01 (-0.57, 0.61) | 0.929 |
|  |  |  |
| **Moderated mediation** | |  |
|  |  |  |
| **Individual SES disadvantages (concurrent disadvantage.)** | | |
| **SEDs at MIDUS 2** | |  |
|  | Estimate | p-value |
| ACME (control) | 0 (0, 0) | 0.549 |
| ACME (treated) | 0 (0, 0) | 0.546 |
| ADE (control) | 0.01 (0, 0.02) | 0.102 |
| ADE (treated) | 0.01 (0, 0.02) | 0.102 |
| Total Effect | 0.01 (0, 0.02) | 0.089 |
| Prop. Mediated (control) | 0.03 (-0.32, 0.52) | 0.583 |
| Prop. Mediated (treated) | 0.04 (-0.32, 0.53) | 0.580 |
| ACME (average) | 0 (0, 0) | 0.547 |
| ADE (average) | 0.01 (0, 0.02) | 0.102 |
| Prop. Mediated (average) | 0.03 (-0.32, 0.53) | 0.581 |
|  |  |  |
| **Individual SES disadvantages (concurrent disadvantage.)** | | |
| **SEDs at MIDUS 2** | |  |
|  | Estimate | p-value |
| ACME (control) | 0 (0, 0) | 0.841 |
| ACME (treated) | 0 (0, 0) | 0.840 |
| ADE (control) | 0 (-0.01, 0.02) | 0.671 |
| ADE (treated) | 0 (-0.01, 0.02) | 0.671 |
| Total Effect | 0 (-0.02, 0.02) | 0.695 |
| Prop. Mediated (control) | 0 (-1.61, 1.51) | 0.950 |
| Prop. Mediated (treated) | 0 (-1.62, 1.51) | 0.950 |
| ACME (average) | 0 (0, 0) | 0.841 |
| ADE (average) | 0 (-0.01, 0.02) | 0.671 |
| Prop. Mediated (average) | 0 (-1.61, 1.51) | 0.950 |
|  |  |  |
| **Individual SES disadvantages (concurrent disadvantage.)** | | |
| **SEDs at MIDUS 2** | |  |
|  | Estimate | p-value |
| ACME (control) | 0 (0, 0) | 0.853 |
| ACME (treated) | 0 (0, 0) | 0.854 |
| ADE (control) | 0 (-0.02, 0.01) | 0.961 |
| ADE (treated) | 0 (-0.02, 0.01) | 0.961 |
| Total Effect | 0 (-0.02, 0.01) | 0.968 |
| Prop. Mediated (control) | 0 (-1.74, 1.6) | 0.965 |
| Prop. Mediated (treated) | 0.01 (-1.73, 1.6) | 0.961 |
| ACME (average) | 0 (0, 0) | 0.854 |
| ADE (average) | 0 (-0.02, 0.01) | 0.961 |
| Prop. Mediated (average) | 0.01 (-1.74, 1.6) | 0.963 |
|  |  |  |
| **Individual SES disadvantages (concurrent disadvantage.)** | | |
| **SEDs at MIDUS 2** | |  |
|  | Estimate | p-value |
| ACME (control) | 0 (0, 0) | 0.948 |
| ACME (treated) | 0 (0, 0) | 0.950 |
| ADE (control) | 0.01 (0, 0.03) | 0.099 |
| ADE (treated) | 0.01 (0, 0.03) | 0.099 |
| Total Effect | 0.01 (0, 0.03) | 0.096 |
| Prop. Mediated (control) | 0 (-0.42, 0.42) | 0.947 |
| Prop. Mediated (treated) | 0 (-0.42, 0.42) | 0.951 |
| ACME (average) | 0 (0, 0) | 0.949 |
| ADE (average) | 0.01 (0, 0.03) | 0.099 |
| Prop. Mediated (average) | 0 (-0.42, 0.42) | 0.949 |

Supplementary Table 2e Lifetime SED-AL-CP

| **Main mediation** |  |  |  |
| --- | --- | --- | --- |
| **Outcome:** | **High Interference Pain** | | |
| **Lifetime disadvantages** | | | |
| **Total SEDs** |  |  | |
|  | Estimate | p-value | |
| ACME (control) | 0 (0, 0) | 0.4614 | |
| ACME (treated) | 0 (0, 0) | 0.4614 | |
| ADE (control) | 0 (0, 0.01) | 0.2618 | |
| ADE (treated) | 0 (0, 0.01) | 0.2618 | |
| Total Effect | 0 (0, 0.01) | 0.2094 | |
| Prop. Mediated (control) | 0.07 (-1.04, 1.38) | 0.566 | |
| Prop. Mediated (treated) | 0.07 (-1.03, 1.38) | 0.566 | |
| ACME (average) | 0 (0, 0) | 0.4614 | |
| ADE (average) | 0 (0, 0.01) | 0.2618 | |
| Prop. Mediated (average) | 0.07 (-1.04, 1.38) | 0.566 | |
| **Outcome:** | **Low Interference Pain** | | |
| **Lifetime disadvantages** | | | |
| **Total SEDs** |  |  | |
|  | Estimate | p-value | |
| ACME (control) | 0 (0, 0) | 0.8236 | |
| ACME (treated) | 0 (0, 0) | 0.8236 | |
| ADE (control) | 0 (-0.01, 0.01) | 0.923 | |
| ADE (treated) | 0 (-0.01, 0.01) | 0.923 | |
| Total Effect | 0 (-0.01, 0.01) | 0.944 | |
| Prop. Mediated (control) | 0 (-2.09, 2.09) | 0.9672 | |
| Prop. Mediated (treated) | 0 (-2.09, 2.09) | 0.9672 | |
| ACME (average) | 0 (0, 0) | 0.8236 | |
| ADE (average) | 0 (-0.01, 0.01) | 0.923 | |
| Prop. Mediated (average) | 0 (-2.09, 2.09) | 0.9672 | |
| **Outcome:** | **3+ Pain** |  | |
| **Lifetime disadvantages** | | | |
| **Total SEDs** |  |  | |
|  | Estimate | p-value | |
| ACME (control) | 0 (0, 0) | 0.691 | |
| ACME (treated) | 0 (0, 0) | 0.691 | |
| ADE (control) | 0 (0, 0.01) | 0.1612 | |
| ADE (treated) | 0 (0, 0.01) | 0.1612 | |
| Total Effect | 0 (0, 0.01) | 0.1332 | |
| Prop. Mediated (control) | 0.04 (-0.84, 0.89) | 0.7286 | |
| Prop. Mediated (treated) | 0.04 (-0.84, 0.89) | 0.7286 | |
| ACME (average) | 0 (0, 0) | 0.691 | |
| ADE (average) | 0 (0, 0.01) | 0.1612 | |
| Prop. Mediated (average) | 0.04 (-0.84, 0.89) | 0.7286 | |
| **Outcome:** | **0-2 Pain** |  | |
| **Lifetime disadvantages** | | | |
| **Total SEDs** |  |  | |
|  | Estimate | p-value | |
| ACME (control) | 0 (0, 0) | 0.938 | |
| ACME (treated) | 0 (0, 0) | 0.938 | |
| ADE (control) | 0 (-0.01, 0.01) | 0.924 | |
| ADE (treated) | 0 (-0.01, 0.01) | 0.924 | |
| Total Effect | 0 (-0.01, 0.01) | 0.933 | |
| Prop. Mediated (control) | 0 (-1.76, 1.79) | 0.979 | |
| Prop. Mediated (treated) | 0 (-1.76, 1.79) | 0.979 | |
| ACME (average) | 0 (0, 0) | 0.938 | |
| ADE (average) | 0 (-0.01, 0.01) | 0.924 | |
| Prop. Mediated (average) | 0 (-1.76, 1.79) | 0.979 | |
|  |  |  | |
| **Moderated mediation** |  |  | |
| **Outcome:** | **High Interference Pain** | | |
| **Lifetime disadvantages** | | | |
| **Total SEDs** |  |  | |
|  | Estimate | p-value | |
| ACME (control) | 0 (0, 0) | 0.785 | |
| ACME (treated) | 0 (0, 0) | 0.786 | |
| ADE (control) | 0 (-0.01, 0.01) | 0.504 | |
| ADE (treated) | 0 (-0.01, 0.01) | 0.504 | |
| Total Effect | 0 (-0.01, 0.01) | 0.521 | |
| Prop. Mediated (control) | -0.01 (-1.27, 1.41) | 0.918 | |
| Prop. Mediated (treated) | -0.01 (-1.27, 1.41) | 0.919 | |
| ACME (average) | 0 (0, 0) | 0.785 | |
| ADE (average) | 0 (-0.01, 0.01) | 0.504 | |
| Prop. Mediated (average) | -0.01 (-1.27, 1.41) | 0.918 | |
| **Outcome:** | **Low Interference Pain** | | |
| **Lifetime disadvantages** | | | |
| **Total SEDs** |  |  | |
|  | Estimate | p-value | |
| ACME (control) | 0 (0, 0) | 0.533 | |
| ACME (treated) | 0 (0, 0) | 0.533 | |
| ADE (control) | 0 (0, 0.01) | 0.350 | |
| ADE (treated) | 0 (0, 0.01) | 0.350 | |
| Total Effect | 0 (0, 0.01) | 0.309 | |
| Prop. Mediated (control) | 0.05 (-1.05, 1.14) | 0.648 | |
| Prop. Mediated (treated) | 0.05 (-1.04, 1.14) | 0.647 | |
| ACME (average) | 0 (0, 0) | 0.533 | |
| ADE (average) | 0 (0, 0.01) | 0.350 | |
| Prop. Mediated (average) | 0.05 (-1.05, 1.14) | 0.648 | |
| **Outcome:** | **3+ Pain** |  | |
| **Lifetime disadvantages** | | | |
| **Total SEDs** |  |  | |
|  | Estimate | p-value | |
| ACME (control) | 0 (0, 0) | 0.912 | |
| ACME (treated) | 0 (0, 0) | 0.912 | |
| ADE (control) | 0 (-0.01, 0.01) | 0.751 | |
| ADE (treated) | 0 (-0.01, 0.01) | 0.751 | |
| Total Effect | 0 (-0.01, 0.01) | 0.736 | |
| Prop. Mediated (control) | 0.02 (-1.85, 1.78) | 0.915 | |
| Prop. Mediated (treated) | 0.02 (-1.85, 1.78) | 0.911 | |
| ACME (average) | 0 (0, 0) | 0.912 | |
| ADE (average) | 0 (-0.01, 0.01) | 0.751 | |
| Prop. Mediated (average) | 0.02 (-1.85, 1.78) | 0.913 | |
| **Outcome:** | **0-2 Pain** |  | |
| **Lifetime disadvantages** | | | |
| **Total SEDs** |  |  | |
|  | Estimate | p-value | |
| ACME (control) | 0 (0, 0) | 0.969 | |
| ACME (treated) | 0 (0, 0) | 0.968 | |
| ADE (control) | 0 (-0.01, 0.01) | 0.401 | |
| ADE (treated) | 0 (-0.01, 0.01) | 0.401 | |
| Total Effect | 0 (-0.01, 0.01) | 0.397 | |
| Prop. Mediated (control) | 0 (-1.08, 1.23) | 0.964 | |
| Prop. Mediated (treated) | 0 (-1.08, 1.23) | 0.965 | |
| ACME (average) | 0 (0, 0) | 0.969 | |
| ADE (average) | 0 (-0.01, 0.01) | 0.401 | |
| Prop. Mediated (average) | 0 (-1.08, 1.23) | 0.965 | |
